# Exosomal and Plasma Non-Coding RNA Signature Associated with Urinary Albumin Excretion in Hypertension

**DOI:** 10.3390/ijms23020823

**Published:** 2022-01-13

**Authors:** Angela L. Riffo-Campos, Javier Perez-Hernandez, Ana Ortega, Olga Martinez-Arroyo, Ana Flores-Chova, Josep Redon, Raquel Cortes

**Affiliations:** 1 Millennium Nucleus on Sociomedicine (SocioMed) and Vicerrectoría Académica, Universidad de La Frontera, Temuco 4780000, Chile; angela.riffo@ufrontera.cl; 2 Department of Computer Science, ETSE, University of Valencia, 46010 Valencia, Spain; 3 Cardiometabolic and Renal Risk Research Group, INCLIVA Biomedical Research Institute, 46010 Valencia, Spain; javier.perez-hernandez@inserm.fr (J.P.-H.); aortega@incliva.es (A.O.); omartinez@incliva.es (O.M.-A.); afloreschova@gmail.com (A.F.-C.); josep.redon@uv.es (J.R.); 4 Departament of Nutrition and Health, Valencian International University (VIU), 46010 Valencia, Spain; 5 T-Cell Tolerance, Biomarkers and Therapies in Type 1 Diabetes Team, Institut Cochin, CNRS, INSERM, Université de Paris, 75014 Paris, France; 6 Internal Medicine Unit, Hospital Clinico Universitario, 46010 Valencia, Spain; 7 CIBER of Physiopathology of Obesity and Nutrition (CIBEROBN), Institute of Health Carlos III, Minister of Health, 28029 Madrid, Spain

**Keywords:** urinary albumin excretion, hypertension, exosomes, plasma, non-coding RNA

## Abstract

Non-coding RNA (ncRNA), released into circulation or packaged into exosomes, plays important roles in many biological processes in the kidney. The purpose of the present study is to identify a common ncRNA signature associated with early renal damage and its related molecular pathways. Three individual libraries (plasma and urinary exosomes, and total plasma) were prepared from each hypertensive patient (with or without albuminuria) for ncRNA sequencing analysis. Next, an RNA-based transcriptional regulatory network was constructed. The three RNA biotypes with the greatest number of differentially expressed transcripts were long-ncRNA (lncRNA), microRNA (miRNA) and piwi-interacting RNA (piRNAs). We identified a common 24 ncRNA molecular signature related to hypertension-associated urinary albumin excretion, of which lncRNAs were the most representative. In addition, the transcriptional regulatory network showed five lncRNAs (LINC02614, BAALC-AS1, FAM230B, LOC100505824 and LINC01484) and the miR-301a-3p to play a significant role in network organization and targeting critical pathways regulating filtration barrier integrity and tubule reabsorption. Our study found an ncRNA profile associated with albuminuria, independent of biofluid origin (urine or plasma, circulating or in exosomes) that identifies a handful of potential targets, which may be utilized to study mechanisms of albuminuria and cardiovascular damage.

## 1. Introduction

Hypertension is a multifactorial disease that affects cardiovascular and renal systems [1,2], and persistently increased urinary albumin excretion (UAE) is a marker of cardiovascular risk progression and renal impairment [3,4]. The mechanisms leading to progression of renal disease and albuminuria are incompletely understood.

Non-coding RNA (ncRNA) species comprise more than 90% of all transcripts and have attracted increasing attention in a broad range of biological processes over the last decade [5,6,7]. NcRNAs can be divided into three categories based on their length: ncRNAs longer than 200 nucleotides (nt), including ribosomal RNA (rRNA) and long non-coding RNA (lncRNA); ncRNAs between 40 nt and 200 nt, such as transfer RNA (tRNA), small nucleolar RNA (snoRNA) and small nuclear ribonucleic acid RNA (snRNA); and ncRNA shorter than 40 nt, such as microRNA (miRNA), piwi-interacting RNA (piRNA) and small interfering (siRNA) [8,9]. NcRNAs’ expression is tissue and cell-type specific under physiological conditions and plays an important role in many biological processes by regulating gene expression at epigenetic, transcriptional and post-transcriptional levels [10,11,12].

Among these macromolecules, the role of miRNAs in the kidney has been studied extensively, and preliminary evidence indicates that they may regulate progression of glomerular and tubular diseases [13,14,15,16]. Current knowledge on lncRNAs has attracted attention over the last few years in both glomerular and tubulointerstitial kidney diseases, such as in diabetic nephropathy [17]. Competitive binding between lncRNAs, target mRNAs and miRNAs is thought to regulate gene expression, forming a wide RNA-based transcriptional regulatory network (lncRNA–miRNA–mRNA) in a broad group of diseases [18,19,20]. Another class of small non-coding regulatory RNAs are piRNA, a recently discovered class whose many aspects in terms of biogenesis, relevance to health and disease, and overall gene regulatory mechanisms remain elusive [21]. Previous evidence suggests that piRNA serve as upstream mediators of epigenetic control and may also be involved in transcriptional gene silencing [22,23].

NcRNAs can be released into circulation bound to RNA-binding proteins or packaged into extracellular vesicles (EVs), such as exosomes, and may function as paracrine effectors in the crosstalk between different cell types in the kidney [24,25]. Recent studies have demonstrated the role of exosomal ncRNAs as biomarkers in urological malignancies, chronic kidney disease, cancer or psoriasis [26,27,28]. Our group has recently found that urinary- and plasma-derived exosomes reveal a distinct miRNA signature associated with albuminuria in hypertension, reflecting changes taking place in the kidney [29]. Nevertheless, a study that analyzes the global ncRNA profile associated with early renal damage in hypertension remains largely unknown.

Our aim was to identify a combined signature of various ncRNA biotypes in liquid biopsy, independent of biofluid origin, in urine, plasma or exosomes from hypertensive patients with albuminuria, using high-throughput sequencing analysis, which may more closely reflect the overall biology of underlying early damage than use of single markers. Finally, we constructed an lncRNA–miRNA–mRNA regulatory network with the ncRNA signature combining bioinformatics and correlation analyses associated with development of UAE in hypertension.

## 2. Results

### 2.1. Characteristics of Study Patients

The study population included 48 essential hypertensive subjects, 22 subjects with increased UAE and 26 normoalbuminurics (non-UAE). General patient characteristics and antihypertensive medication are shown in Table 1.

### 2.2. Proportions of RNA Types in Each Biological Fraction and Patient Groups

Small RNA-sequencing single-end technology was used to detect RNA types in the three different biofluid fractions (total plasma, urinary and plasma-derived exosomes) from hypertensive patients with or without increased UAE. When we analyzed all mapped reads as a whole, we observed that the most frequent RNA biotypes in proportion were piRNA with 38%, miRNA with 32% and miscellaneous RNA (miscRNA) with 16%. This last group included Y-RNA and Vault-RNA, where Y-RNA represented 99% of mapped reads. LncRNA represented 63% of other mapped read groups (Supplemental Material, Figure S1A). In addition, when all genes included in the analysis were examined and separated by RNA type, those encoding small fragments of RNA showed the highest variety of genes (a total of 10,603), followed by lncRNA (718), piRNA (293) and miRNA (159) (Figure S1B).

**Figure 1:**
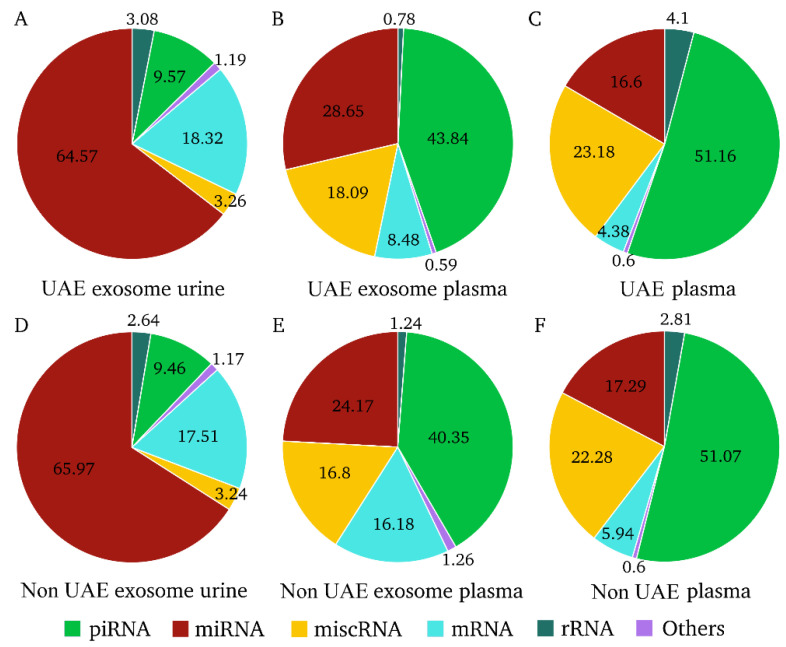
Proportions of RNA types in each biological fraction and patient group. The unit used was filtered, normalized and counts per million (CPM) mapped reads annotated. (**A**–**C**) represent each biological fluid in hypertensive patients with urinary albumin excretion (UAE). (**D**–**F**) represent each biological fluid in non-UAE patients. miscRNA: miscellaneous RNA; miRNA: microRNA; mRNA: messenger RNA; piRNA: PIWI-interacting RNA; rRNA: ribosomal RNA.

We next sought to analyze the proportion of RNA biotypes present in each of the three biological biofluids, finding miRNA to be the predominant biotype with mapped reads in urinary exosome fraction, representing approximately 65% of total mapped reads in both hypertensive patient types (with or without UAE). Nonetheless, piRNAs were also the most representative RNA type in both patient groups, with 40% in plasma exosomes and 51% in plasma (Figure 1). Of the remaining RNA types identified, mRNA, rRNA, miscRNA and others (where lncRNA represented between 75% to 90% of reads) showed similar percentages in the three biofluids, regardless of the presence of UAE. These data indicate that biofluid origin, mainly if stemming from urine or plasma samples, influences RNA type distribution. In addition, non-significant differences were observed in all biofluids when comparing patient groups with and without UAE.

### 2.3. Differentially Expressed RNAs in Microalbuminuria in Each Biological Fraction

**Figure 2:**
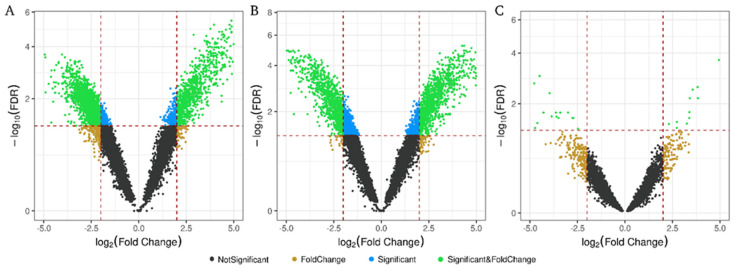
Differentially expressed RNAs in hypertensive patients with UAE in each biological fraction. Volcano plot depicts significantly altered RNAs found in (**A**), plasma exosomes; (**B**), urinary exosomes; and (**C**), in plasma. Each dot represents an RNA; non-significant false discovery rate (FDR > 0.05) and log_2_ fold-change ≤2 or ≥−2) in black, with log_2_ fold-change ≥2 or ≤−2 in brown, with significant FDR in blue and with significant FDR and log_2_ fold-change ≥2 or ≤−2 in green. The threshold dotted line for log_2_ fold-change was ≤2 or ≥−2, and for −log_10_(FDR) it was <0.05.

As shown in the volcano plot (Figure 2), analysis of RNA subtypes in all patients for each biofluid identified more significant RNAs differentially expressed (DE) (FDR < 0.05) in exosome fraction than in plasma, regardless of whether exosomes came from urine or plasma. Significant RNA showed higher fold change (FC) and FDR in urinary and plasma exosomes than in those circulating in plasma (Figure 2A,B vs. Figure 2C). Analyzing the number of DE transcripts (considering *p*-value < 0.05), 4336 were found in urinary exosomes (U-Exo), 4645 in plasma exosomes (*p*-Exo) and 1415 in plasma samples (Supplemental Material, Figure S2A). In the three biofluids, the protein-coding genes correspond to approximately 85% of the total transcripts (Figure S2B), followed by 7% lncRNA and 2% miRNA. Interestingly, transcript type distributions obtained among the biological compartments showed very limited overlapping between exosomal and plasma fractions, with only 199 out of 10,396 common to all three groups (Supplemental Material, Figure S2A), of which 88% were protein-coding genes (175 transcripts).

### 2.4. Differentially Expressed Non-Coding RNAs by Origin

**Figure 3:**
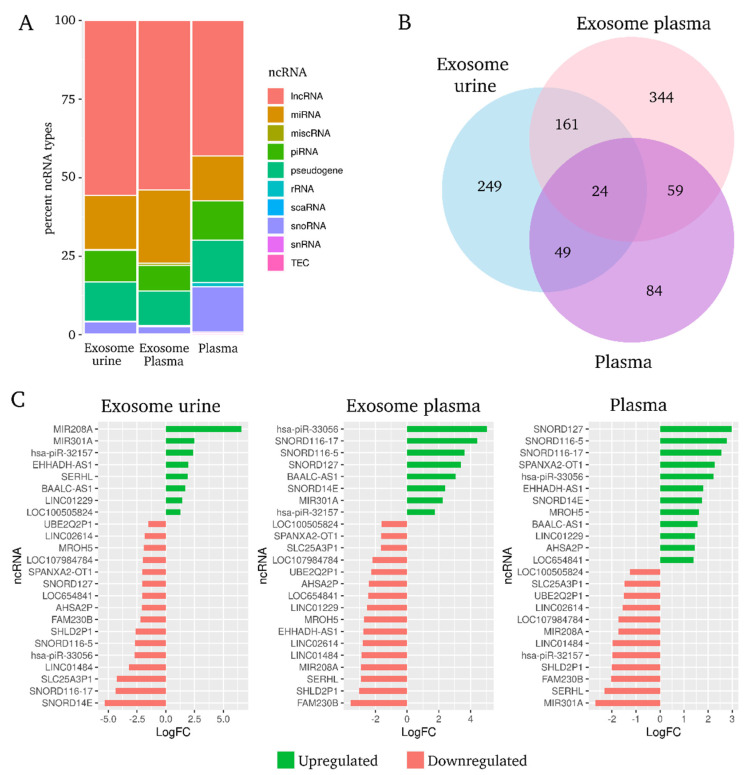
Differentially expressed non-coding RNAs by biofluid origin. (**A**), Characterization of differentially expressed non-coding RNA type in hypertensive patients with UAE in exosome urine, exosome plasma and plasma biofluids. (**B**), Venn diagram shows the overlap among biological fractions. (**C**), Diverging bar charts give the fold-change expression of the 24 common non-coding RNAs in each biological fraction: upregulated are in green and downregulated in red. logFC: logarithm 2 base fold change; lncRNA: long non-coding RNA; miRNA: microRNA; miscRNA: miscellaneous RNA; piRNA: PIWI-interacting RNA; rRNA: ribosomal RNA; scaRNA: small Cajal body-specific RNA; snRNA: small nuclear RNA; snoRNA: small nucleolar RNA; TEC: to be experimentally confirmed.

Analyzing DE ncRNA among hypertensive patients with or without UAE, we observed that the three RNA biotypes with the greatest number of statistically significant transcripts were lncRNA (52% in exosome fractions and 44% in plasma), miRNA (20% in U-Exo, 26% in P-Exo and 13% in plasma) and piRNA (15% in U-Exo and plasma, 10% in P-Exo) (Figure 3A). Next, the Venn diagram obtained from among the biological compartments showed very limited overlapping between exosomal and plasma fractions, with 24 of these 835 DE ncRNAs common to all three groups. Ten of them were lncRNA (42%), six pseudogenes (25%), four snoRNAs (17%), two miRNAs (8%) and two piRNAs (8%). These 24 ncRNAs represent the molecular signature related with hypertension-associated UAE, independent of biofluid (U-Exo, P-Exo or circulating in plasma) (Figure 3B).

Diverging bar charts showed the fold-change expression of the 24 common ncRNAs in each biological fraction (Figure 3C). The majority of lncRNAs were downregulated in hypertensive patients with UAE in all three biofluids. In both urine and plasma exosome fractions, hsa-piR-32157 was upregulated and downregulated in plasma samples, and the other hsa-piRNA-33056 was upregulated in P-Exo and plasma but downregulated in U-Exo from patients with UAE. Likewise, both miRNAs (miR-208a and miR-301a) were significantly augmented in U-Exo, despite being highly downregulated in plasma fraction. All four snoRNAs were downregulated in U-Exo fraction but were upregulated in plasma. Finally, the vast majority of pseudogenes were downregulated in both exosome and plasma fractions from hypertensive patients with UAE (Figure 3C).

### 2.5. Common Differentially Expressed lncRNA–miRNA–mRNA Network from Hypertensive Patients with Urinary Albumin Excretion

**Figure 4:**
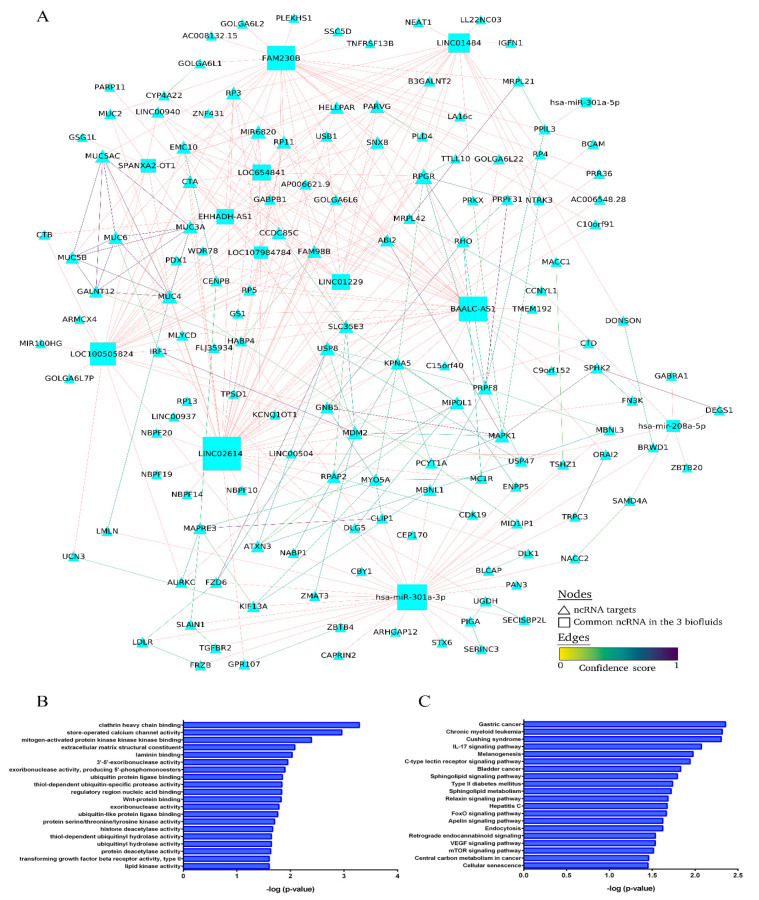
Overview of the common lncRNA–miRNA–mRNA target interaction network related to hypertension-associated UAE. (**A**), Each common ncRNA in the three biofluids is a square node; ncRNA targets are triangle nodes. The node size increases in relation to the number of edges (network degree), and a higher confidence score indicates a stronger edge between nodes. The predicted interaction between the ncRNA and its target is shown in pink, representing the same confidence for all cases. (**B**), The top 20 most significant Gene Ontology terms. (**C**), The top 20 most significant Kyoto Encyclopedia of Genes and Genomes (KEGG) pathway terms.

The ten DE lncRNAs and two miRNAs established in the molecular signature were selected and potential predicted target mRNAs were identified, creating the common DE lncRNA–miRNA–mRNA network (Figure 4). Hub nodes, characterized by their high degree of connectivity to other nodes in the network, can be used to assess the significance of genes in the network. In the present study, five lncRNAs (LINC02614, BAALC-AS1, FAM230B, LOC100505824 and LINC01484) and one miRNA (miR-301a-3p) were observed to be topological hub nodes whose betweenness, network degree and closeness centrality were significantly higher in comparison with other common RNAs (Table 2). In addition, the Over-Representation Analysis using GO annotation showed that clathrin heavy chain binding, store-operated calcium channel activity, mitogen-activated protein kinase (MAPK) binding and extracellular matrix structural constituent were among other pathways that could play an important role in development of albuminuria, but more evidence is necessary. The Over-Representation Analysis further reported that among the most significant pathways were IL-17 signaling, sphingolipid signaling and metabolism, type II diabetes mellitus, endocytosis and vascular endothelial growth factor (VEGF) signaling (Figure 4B,C).

### 2.6. Protein–Protein Interaction Network of Differentially Expressed mRNA in Common to All Biofluids Associated with Albuminuria

To further clarify the biological roles of the common short fragments of protein-coding DE RNA in the three biofluids from hypertensive patients with UAE, we performed the gene set Over-Representation Analysis (ORA) with the following findings: 144 common transcripts in the three biofluids and four networks related to pathogenesis of hypertension and presence of UAE were identified, showing a central node with more than six edges.

**Figure 5:**
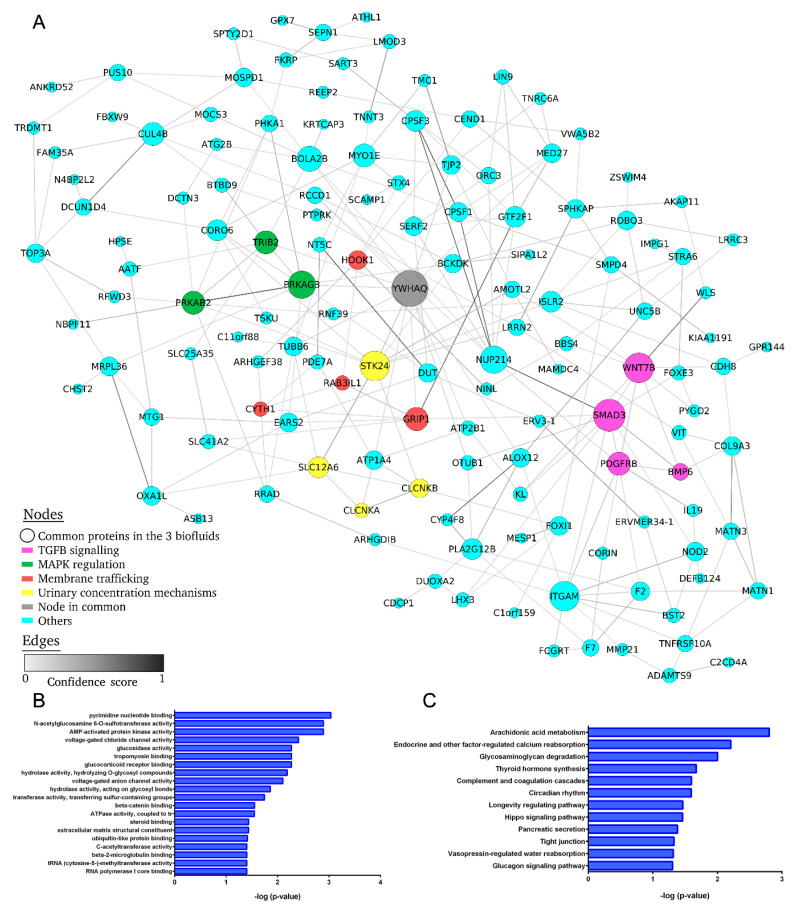
Protein–protein interaction network of common differentially expressed mRNAs in all biofluids in microalbuminuria patients. (**A**), Each common mRNA in the tree biofluids is a node (circle); edges indicate direct interactions between nodes, the node size increases according to the number of edges (network degree), and a higher confidence score indicates a stronger edge between nodes. Four main sub-networks related with the pathogenesis of hypertension and the presence of UAE were identified: TGF-β signaling (pink), MAPK regulation (green), membrane trafficking (red) and urinary concentration mechanisms (yellow). (**B**), The top 20 most significant Gene Ontology terms. (**C**), The significant Kyoto Encyclopedia of Genes and Genomes (KEGG) pathway terms.

The first network identified is associated with transforming growth factor (TGF)-signaling and includes SMAD3, WNT7B, BMP6 and PDGFRB proteins. The second important network is associated with kidney urinary concentration mechanisms, such as kidney water reabsorption, salt reabsorption and K/Cl cotransporter (YWHAQ, STK24, SLC12P6, CLCNKA and CLCNKB proteins). The third network is linked to modulation of MAPK signaling, including YWHAQ, PRKAG3, PRKAB2 and TRIB2 proteins. Finally, the last network mediates membrane trafficking with YWHAQ, GRIP1, RAB3IL1, HOOK1 and CYTH1 proteins involved (Figure 5). In addition, the DE mRNA-related GO analysis showed that voltage-gated chloride and anion channel activity, glucocorticoid receptor binding, extracellular matrix (ECM) structured constituent and others could play an important role in development of albuminuria. The pathway analysis further revealed that 12 unique pathways were enriched, including the factor-regulated calcium reabsorption, complement and coagulation cascades, tight junction and vasopressin-regulated water reabsorption.

Finally, we generated a new interaction network joined to the lncRNA–miRNA–mRNA targets interaction network (Figure 4), with the protein–protein interaction network of common DE mRNA (Figure 5). The new network showed numerous interactions between the nodes of the two sub-networks, mainly at secondary level (Figure S3). Among the 19 DE genes with node degree >10, we found nine of the ten common lncRNAs, one common miRNA and ten protein-coding genes (Table S1).

## 3. Discussion

In the present study, we identified a 24-ncRNA signature associated with albuminuria in hypertension, independent of biofluid, which is common to urinary exosomes, plasma exosomes and circulating in plasma, containing predominantly lncRNA. We also constructed a transcriptional regulatory network (common lncRNA–miRNA–mRNA targets) and predicted the target genes. We found five lncRNAs (LINC02614, BAALC-AS1, FAM230B, LOC100505824 and LINC01484) and one miRNA (miR-301a-3p) with significantly higher node degree and topological network values compared with the other nodes, implying that these hub RNAs are essential in network organization and are potential key regulators controlling UAE development in hypertension-related RNA network. In addition, we used Gene Ontology (GO) and Kyoto Encyclopedia of Genes and Genomes (KEGG) pathway analysis to assess the enriched biological functions regulated by the ncRNA signature, most of them implicated in mechanisms inducing renal damage.

A key feature of the present study was the strategy used. The vast majority of previous studies have identified a unique ncRNA biotype, mainly miRNA, associated with hypertension renal damage in a specific biological fraction [30,31], whereas our multicompartment approach aimed to provide a common global ncRNA profile associated with albuminuria, independent of sample origin. By combining the differentially expressed ncRNAs found in total plasma, urinary and plasma-derived exosomes, we sought to find an ncRNA signature representative of albuminuria development in hypertension that could be identified in clinical practice, regardless of the type of patient sample available (urine, plasma or exosomes). We observed that the behavior of ncRNA levels differed from circulating in plasma or in exosomes, several of them being downregulated in plasma and upregulated in exosomes and vice-versa. Furthermore, common ncRNA level changes were dependent on urine or plasma origin. Previous studies found selective sorting of specific miRNAs into exosomes compared with the whole miRNA circulating pool in specific biological fractions [32,33]. Our data suggest that an increase in exosomal ncRNA expression levels to the detriment of circulating levels could be due to a controlled, specific process related to a pathological condition. These findings identify a common ncRNA profile in all three fractions, which facilitates its identification in clinical practice, independent of sample origin, and biofluid origin will only take into account the interpretation of albuminuria-related expression changes (up- or down-regulation).

Analyzing DE ncRNA in hypertensive patients with or without UAE, the three most representative ncRNA biotypes were lncRNA, miRNA and piRNA in all three biofluids. Considerable research has been conducted over recent years into the molecular mechanisms of hypertension-associated renal pathology; however, most previous studies have focused mainly on protein-coding genes or miRNAs [30,31,34,35,36]. For example, our group revealed an exosomal miRNA signature associated with albuminuria in hypertension [29]. The lncRNA group has reached special relevance in the last years in health and diseases, but few studies have reported on the role of lncRNA in renal pathology [12,17]. In recent years, another ncRNA group, piRNAs, have gained prominence as modulators of disease pathogenesis. A number of studies have reported that piRNA dysregulates expression in samples of different diseases, and various potential mechanisms have been proposed [22,37]. The present study contributes significantly to the literature due to our global analysis of ncRNA profile in hypertensive patients to assess an ncRNA signature related to albuminuria.

The common 24-ncRNA profile related to albuminuria in hypertension was composed mostly of lncRNA, followed, in order, by pseudogenes, snoRNAs, miRNAs and piRNAs. Ten DE lncRNAs were identified and in the RNA network constructed, five of these showed the highest node degree and significant role in interacting with ncRNA targets, serving as hub nodes (LINC02614, BAALC-AS1, FAM230B, LOC100505824 and LINC01484) joined to miRNA-301a. The GO and KEGG pathway analyses assessing biological functions enriched in response to albuminuria highlighted several pathways: modulators in the renal epithelial–mesenchymal transition (MAPK binding, TGF-β receptor and IL-17 signaling pathway), renal fibrosis (Wnt-protein binding, sphingolipid signaling and metabolism), endocytosis (clathrin heavy chain binding and store-operated calcium channel (SOCC) activity) and ECM constituent (SOCC activity and laminin binding). Accumulating experimental evidence indicates that these enriched pathways have always been involved in renal impairment. As an example, Chaudhari et al. emphasized SOCC as a crucial regulator of ECM synthesis and deposition by glomerular mesangial cells, its dysregulation being implicated in the pathogenesis of mesangial and interstitial fibrosis in diabetic nephropathy [38,39,40,41]. Furthermore, four snoRNAS related to albuminuria in hypertension were identified in the signature. A specific genome-wide array analysis of SNORD116 cluster showed that this small RNA changed the mRNA expression levels of over 200 genes, which was associated with clinical findings [42]. Finally, two piRNAs also showed altered expression levels in the common ncRNA signature. PiR-33056 has the guanine nucleotide exchange factor VAV3 as potential target gene, which is associated with the nucleotide-free states of Rho GTPases that activate pathways leading to actin cytoskeletal rearrangements [43]. As a result of these above-mentioned analyses, we identified an ncRNA signature and molecular network that could play an important role, by way of these pathways, in the development of hypertension-associated albuminuria.

Additionally, we predicted a common protein-coding gene network for the three biofluids, finding among the nodes with highest degree several transcripts known to be associated with albuminuria development and progression of kidney damage in hypertension, such as: SMAD3, WNT7B, BMP6 and PDGFRB (TGF-β signaling) [44]; YWHAQ, STK24, SLC12P6, CLCNKA and CLCNKB (kidney urinary concentration mechanisms) [45]; YWHAQ, PRKAG3, PRKAB2 and TRIB2 (MAPK regulation); and YWHAQ, GRIP1, RAB3IL1, HOOK1 and CYTH1 (membrane trafficking) [46,47]. Next, the GO terms and enriched KEGG pathways revealed voltage-gated chloride and anion channel activity, vasopressin-regulated water reabsorption, complement and coagulation cascades and glucocorticoid receptor binding as mechanisms related to renal impairment in hypertension. An extended body of evidence supports the existence of pathways obtained in this study, some of which occur at the glomerulus (podocytes, endothelial cells) and others in the renal tubules. For example, ion channel (CLCNKA and CLCNKB) and transporter (SLC12P6) alteration, which act in concert to regulate volume and ionic concentration by absorption or secretion of ions into the urine, leads to renal disease [48,49]. Srivastava et al. demonstrated that loss of podocyte glucocorticoids receptors leads to upregulation of Wnt signalling and disruption in fatty acid metabolism, important for glomerular homeostasis [50]. The most striking feature of tubulointerstitial fibrosis is excessive deposition of fibrillar material in the widened interstitial space in fibrotic kidneys, and the condition is characterized by production of fibrosis-promoting factors, such as TGF-β1 and PDGF [41], both identified in our protein-coding network. Finally, previous works have shown that systemic endothelial dysfunction is an initiating step in the development of vascular damage, and albuminuria reflects widespread vascular damage [51], being a prognostic factor for cardiovascular risk in hypertension [4]. Therefore, plasma ncRNA signature associated to albuminuria could also indirectly reflect the cardiovascular risk progression in albuminuric hypertensive patients.

The major goal of this study was to identify a combined signature of various ncRNAs, such as lncRNAs, miRNAs and piRNAs, independent of biofluid origin, which may more closely reflect the overall biology of underlying early damage in hypertension than use of single markers. Another highlight was to assess the ncRNA targets to construct a regulatory network and identify the hub nodes that play an important role in network organization. These findings provide insights into the mechanisms involved in the architecture of the glomerular filtration barrier and renal tubular reabsorption and provide potential targets for treating hypertension-associated albuminuria. As discussed above, these data are supported by literature evidence in association with renal impairment. Hence, experimental validation of these findings, identifying precise cellular sources and mechanisms underlying common ncRNAs, are expected to set a benchmark for early renal damage research. Finally, further research using larger and independent cohorts is warranted to confirm the ncRNA signature found.

In summary, our study found an ncRNA profile associated with albuminuria, independent of biofluid origin (urine or plasma, in exosomes or circulating), that targets critical pathways of filtration barrier integrity, tubule reabsorption and vascular endothelial function, suggesting an important role for ncRNA signature in hypertension-associated early renal damage and cardiovascular risk progression. Further experimental studies should be performed to demonstrate the utility of these candidates as promising therapeutic targets in albuminuria and widen opportunities in comprehensive renal damage research.

## 4. Materials and Methods

### 4.1. Subjects

This was an observational case-control study which included 21 patients with essential hypertension (*n* = 21) and 22 patients without persistent elevated urinary albuminuria (UAE) (≥30 mg/g urinary creatinine) [52]. All hypertensive patients received antihypertensive treatment at the time of the study, and the mean duration of disease progression was five years. Hypertensive patients with severe kidney disease, uncontrolled hypertension, resistant hypertension or secondary hypertension, were excluded. The samples correspond to small RNA-Seq single-end raw data from three different biofluid fractions (total plasma, urinary and plasma-derived exosomes).

### 4.2. Biological Samples

Fresh first morning urinary samples (100 mL) were collected in sterile containers, and human blood samples were collected in EDTA tubes and centrifuged to separate the plasma fraction. All samples were processed within one hour after reception to isolate the exosomal component, as explained below.

### 4.3. Exosome Isolation and Characterization

Exosomes were isolated from urine (Exo-U) and plasma (Exo-P), using a protocol based on sequential ultracentrifugation. Exosome pellets were characterized by qNano Gold instrument (Izon Science Ltd., Christchurch, New Zealand), transmission electron microscopy and western blot. Detailed protocols are explained in our previous study authored by Perez-Hernandez J et al. [29].

### 4.4. RNA Extraction, Small RNA Library Preparation and Next-Generation Sequencing

Total RNA was extracted from exosomes using the Total Exosome RNA and Protein Isolation kit (Invitrogen, Life Technologies, Carlsbad, CA, USA); RNA was obtained using the miRNeasy mini kit (Qiagen, Hilden, Germany) from plasma samples. Quantification of total RNA, quality and size distribution were analyzed by capillary electrophoresis (Agilent 2100 Bioanalyzer, Agilent Technologies, Santa Clara, CA, USA) with the RNA 6000 Pico chip.

Single-patient libraries were prepared from 2 μL of total RNA from each condition (total plasma, urinary exosomes or plasma exosomes) using CleanTag Small RNA library preparation kit (TriLink Biotechnologies, San Diego, CA, USA), following a small RNA library preparation protocol optimized to very low input samples, as previously described [53]. Libraries were sequenced on the HiSeq 2000 platform (Illumina, San Diego, CA, USA) at 8 pM final concentration with a 50-cycle single-read mode (CNAG, Barcelona, Spain). The raw RNA-Seq dataset is available at the BioProject repository, accession: PRJNA590749.

### 4.5. Small RNA Sequencing Data Analysis

**Figure 6:**
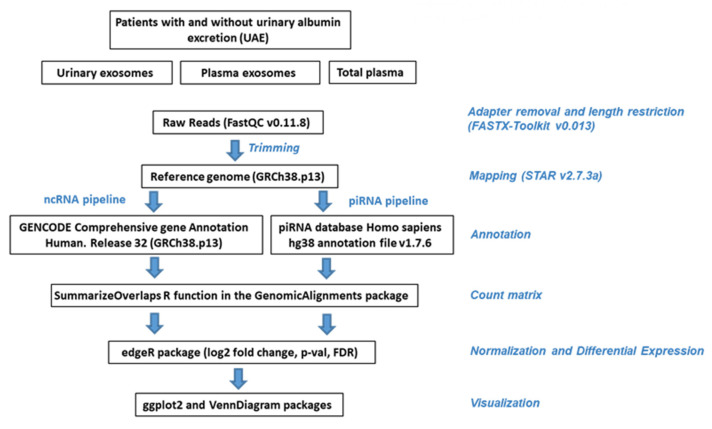
Schematic workflow representing the study methodology.

Data quality control of the raw data was conducted with FastQC v0.11.8 [54]. Subsequently, the data was filtered using FASTX-Toolkit v0.013 (http://hannonlab.cshl.edu/fastx_toolkit, accessed on 13 October 2021), removing adapters, low quality reads and nucleotides. Alignment was made with STAR v2.7.3a [55], following the recommendations for use in small RNA-Seq data. GENCODE human genome release 38 (GRCh38.p13) was used as a reference genome. SAM files were converted to BAM and sorted with SAMtools v1.10 [56]. Sorted BAM files were included in R, and the count matrix was obtained using GenomicFeatures [57], Rsamtools [58] and GenomicAlignments Bioconductor packages. Two gtf annotation files were used: GENCODE reference annotation for the Human release 38 (comprehensive gene annotation) [59] and piRNA database Homo sapiens hg38 annotation file v1.7.6 [60]. We therefore followed two pipeline analyses: one for ncRNA and the other for piRNA (Figure 6 and Supplementary Methods File).

### 4.6. Preprocessing, Annotation and Normalization

The count matrix was included in a DGEList object using the edgeR Bioconductor package [61]. The metadata for samples was included, and the genes not expressed in either experimental condition were discarded. Annotation was performed by org.Hs.eg.db Bioconductor package [62], which provides a genome-wide annotation for Human (see Figure 6 and Supplementary Material Methods). A matrix with filtered, normalized and annotated counts per million (CPM) mapped reads was generated for piRNA pipeline and another one for ncRNA pipeline to estimate the abundance of RNA types in each group of samples, summing up the counts of these two matrices (Supplementary Methods file).

### 4.7. Statistical Analysis

The contrasts between hypertensive patients with (UAE) and without albuminuria (non-UAE) were determined by performing a negative binomial generalized log-linear model to analyze the read counts for each gene, adjusted for sex. The *p*-values were adjusted using Benjamini–Hochberg method. *p* < 0.05 was considered statistically significant. The edgeR Bioconductor package was used for all statistical analyses [61]. The graphs were made using the R package ggplot2 [63] or VennDiagram [64], as appropriate (Supplementary Methods File).

### 4.8. Non-Coding RNA Target Predictions

The targets for lncRNAs were predicted using LncRRIsearch, a web server for comprehensive prediction of human and mouse lncRNA–lncRNA and lncRNA–mRNA interaction (http://rtools.cbrc.jp/LncRRIsearch, accessed on 13 October 2021) [65]. The top ten targets for each isoform with an energy threshold ≤−100 kcal/mol were selected for each lncRNA gene.

In the case of miRNA-targets, three web-based tools were used: TargetScan (http://www.targetscan.org/vert_72/, accessed on 13 October 2021) [66], miRDB (http://mirdb.org/, accessed on 13 October 2021) [67] and miRTarBase (https://mirtarbase.cuhk.edu.cn/~miRTarBase/miRTarBase_2022/php/search.php, accessed on 13 October 2021) [68]. The selection criteria by target was a cumulative weighted context++ score of <−0.5 for TargetScan. For miRTarBase, the targets were selected with a number of papers greater than 1 or if the sum of validation methods was greater than 1. For miRDB, all targets with a Target Score of 90 or higher were selected. The targets predicted in common for at least two tools were selected, except for hsa-mir-208a-5p, where only miRDB predicted targets, i.e., all targets with a Target Score of 99 or higher, were selected.

### 4.9. Molecular Pathways Analyses

Gene set Over-Representation Analysis (ORA) was performed in WEB-based GEne SeT AnaLysis Toolkit (http://www.webgestalt.org/, accessed on 15 October 2021), using GO and KEGG databases [69]. The protein–protein interaction network was generated using STRING database v11.0 [70]. All biological interactions with a confidence score of 0.2 or greater were included. The STRING database provides a confidence score (from 0 to 1), which estimates the likelihood that an annotated interaction between a pair of proteins is biologically meaningful, specific and reproducible. The networks were analyzed and displayed using the yFiles organic layout with Cytoscape v3.8.1 [71]. In this network, nodes and edges represented biological data in a direct manner, in which each node represented a biological molecule, and the edges represented interactions between nodes. The ncRNA-target network was generated using STRING to obtain the interaction between targets, following the same methodology as for the protein network. LncRNA-target and miRNA-target interaction was included using Cytoscape, based on predictions by the web-based tools described above (Section 4.9), using a manually generated sif file.

## Figures and Tables

**Table 1:** Clinical characteristics of hypertensive patients by study group.

Variables	Albuminuria (UAE) (*n* = 22)	Normoalbuminuria (Non-UAE) (*n* = 26)
Age (years)	52.2 ± 8.3	55.0 ± 5.3
Gender (male)	68.2%	65.4%
SBP (mmHg)	136± 15	136 ± 24
DBP (mmHg)	85 ± 10	87 ± 14
PP (mmHg)	51 ± 12	48 ± 17
Glucose (mg/dL)	122 ± 46	119 ± 41
Glycated hemoglobin (%)	6.6 ± 1.2	6.0 ± 0.9
Total cholesterol (mg/dL)	200 ± 34 **	173 ± 29
LDL (mg/dL)	128 ± 30 **	108 ± 25
HDL (mg/dL)	51 ± 14	50 ± 10
Triglycerides (mg/dL)	153 ± 78	127 ± 60
Plasma creatinine (mg/dL)	0.87 ± 0.30	0.90 ± 0.22
GFR (mL/min/1.73 m^2^)	96 ± 27	87 ± 19
Body mass index (kg/m^2^)	32 ± 7	30 ± 5
Obesity grade (%)		
Grade I	29	20
Grade II	9	12
Grade III	14	8
Diabetes (%)	41	35
Dyslipidemia (%)	86	85
Smoking (%)	55	48
UAE/Creatinine (mg/g)	146.4 ± 144.3 ***	3.1 ± 1.7
Antihypertensive treatment (%)		
ARB	95	92
CCB	36	38
Diuretics	68	62
Statins	32	8

ARB: angiotensin receptor blockers; CCB: calcium channel blockers; DBP: diastolic blood pressure; eGFR: estimated glomerular filtration rate; PP: pulse pressure; SBP: systolic blood pressure; UAE: urinary albumin excretion. ** *p* value < 0.001; *** *p* value < 0.0001.

**Table 2:** List of common transcripts in the lncRNA–miRNA–mRNA network with high degree node.

RNA	Degree	Betweenness Centrality	Closeness Centrality
LINC02614	49	0.321215546	0.447368421
hsa-miR-301a-3p	34	0.223753645	0.354166667
BAALC-AS1	32	0.127310962	0.392307692
FAM230B	31	0.136936472	0.375
LOC100505824	28	0.128194316	0.387341772
LINC01484	20	0.090634319	0.350114416
LOC654841	14	0.020020697	0.348519362
LINC01229	14	0.015796606	0.334792123
EHHADH-AS1	13	0.036008241	0.34537246
SPANXA2-OT1	9	0.01894646	0.31875
LOC107984784	7	0.003982221	0.330453564
hsa-mir-208a-5p	6	0.037362168	0.263339071

## Data Availability

The raw RNA-Seq dataset is available at the BioProject repository, accession: PRJNA590749.

## Supplementary Materials

The following are available online at https://www.mdpi.com/article/10.3390/ijms23020823/s1.
