# Supplementary material for: Exosomal and Plasma Non-Coding RNA Signature Associated with Urinary Albumin Excretion in Hypertension
Source: Int J Mol Sci. 2022 Jan 13;23(2):823. doi: 10.3390/ijms23020823 (PMC8775608; doi:10.3390/ijms23020823)
Supplement: Supplementary file 1 [file ijms-23-00823-s001.zip › ijms-1511157_ Revised_Supplemental material.pdf]

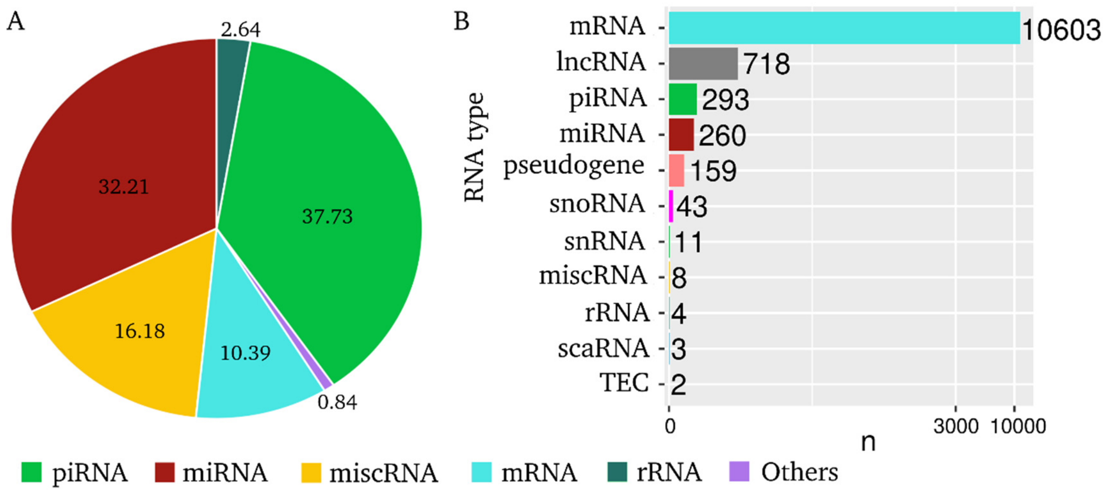

Figure S1. Proportion of RNA types included in the analysis. A) Proportion of filtered, normalized and annotated counts per million (CPM) mapped reads included by RNA type. B) Total genes included in the analysis, separated by encoded RNA type. lncRNA: long non-coding RNA; miscRNA: miscellaneous RNA; miRNA: microRNA; mRNA: messenger RNA; piRNA: PIWI-interacting RNA; rRNA: ribosomal RNA; scaRNA: small cajal body-specific RNAs; snoRNA: small nucleolar RNA; snRNA: small nuclear RNA; TEC: to be experimentally confirmed.

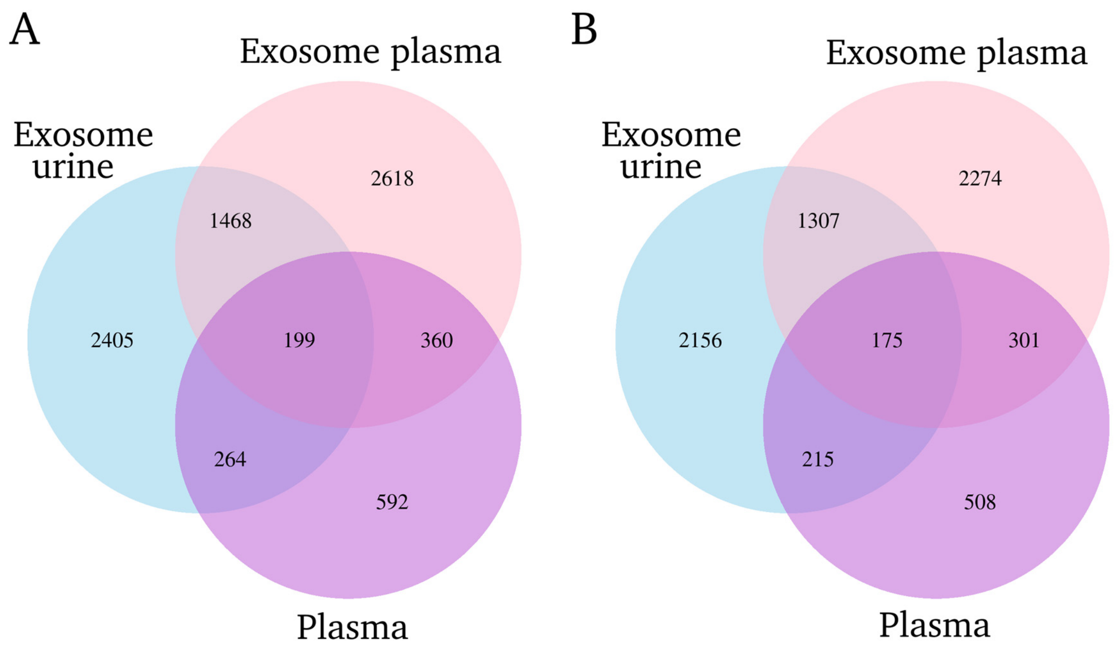

Figure S2. Differentially expressed RNAs according to their biofluid of origin. A) All differentially expressed RNA types. B) Only differentially expressed protein-coding genes.

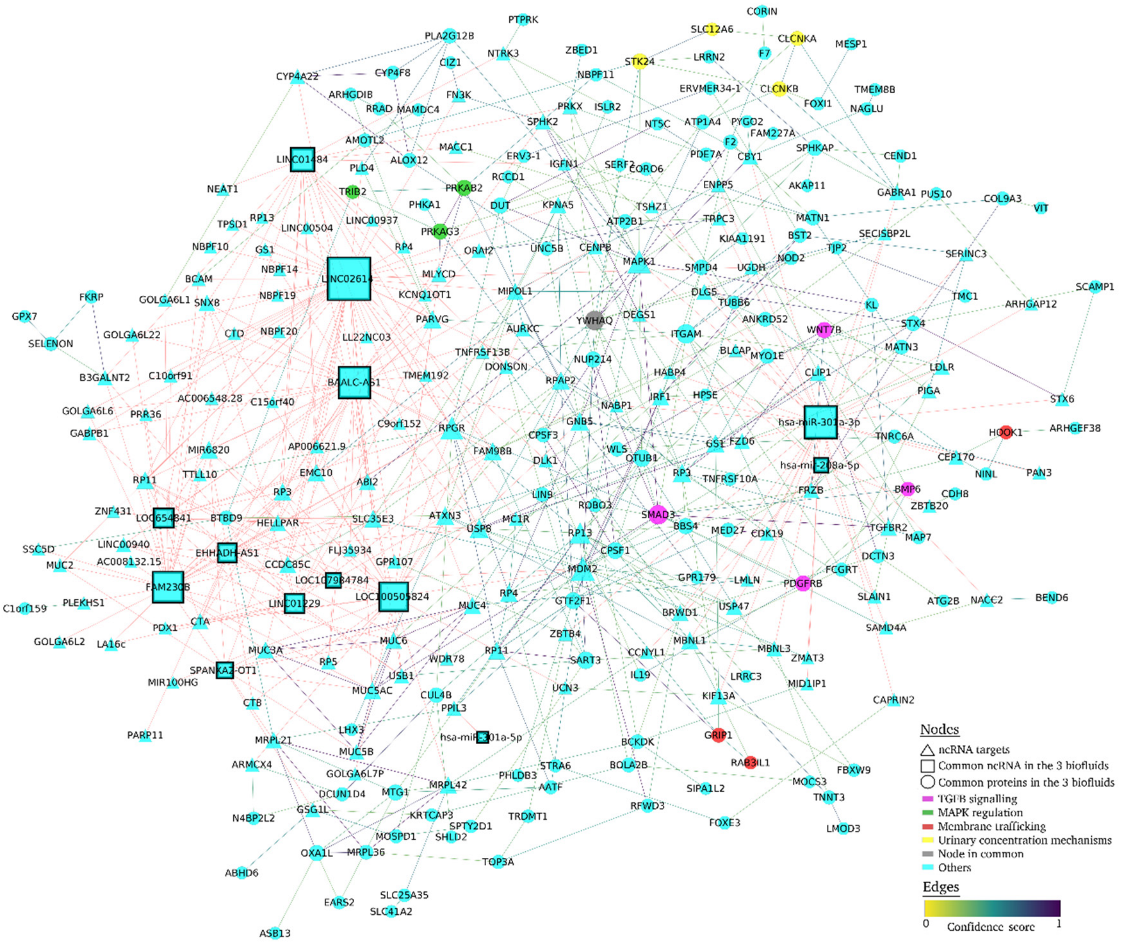

Figure S3. Interaction network joined to the lncRNA-miRNA-mRNA targets with the protein-protein networks of common mRNA differentially expressed in albuminuric patients. Each common protein-coding transcript in the tree biofluids is a node (circle), ncRNA is a square node, and ncRNA targets are triangle nodes; edges indicate direct interactions between nodes, node size increases according to the number of edges (network degree) and a higher confidence score indicates a stronger edge between nodes. Four main protein-coding sub-networks related to pathogenesis of hypertension and presence of UAE were identified: TGF-β signaling (pink), MAPK regulation (green), membrane trafficking (red) and urinary concentration mechanisms (yellow).

Table S1. List of the common transcripts in lncRNA-miRNA-mRNA interaction and protein-coding networks with high node degree

RNAs	Degree	Betweenness centrality	Closeness centrality
LINC02614	49	0.17958105	0.36164736
hsa-miR-301a-3p	34	0.19254103	0.34993773
BAALC-AS1	32	0.07564141	0.33372922
FAM230B	31	0.09671403	0.32865497
LOC100505824	28	0.10889687	0.34906832
LINC01484	20	0.06435499	0.3167982
MDM2	19	0.08636736	0.35750636
RPGR	17	0.09850797	0.37466667
MAPK1	17	0.14423659	0.3707124
RP13	14	0.05069488	0.33452381
LINC01229	14	0.01320194	0.29957356
LOC654841	14	0.01115039	0.30247578
EHHADH-AS1	13	0.01659321	0.29989328
YWHAQ	11	0.05364584	0.34268293
ITGAM	11	0.02340773	0.30477223
SMAD3	11	0.02718376	0.33136792
USP8	11	0.03767062	0.35168961
RP3	10	0.01825634	0.30150215
GNB5	10	0.04557469	0.33019976
