# Supplementary material for: Exosomal and Plasma Non-Coding RNA Signature Associated with Urinary Albumin Excretion in Hypertension
Source: Int J Mol Sci. 2022 Jan 13;23(2):823. doi: 10.3390/ijms23020823 (PMC8775608; doi:10.3390/ijms23020823)
Supplement: Supplementary file 1 [file ijms-23-00823-s001.zip › ijms-1511157_Supplementary Methods.pdf]

Supplementary Material Methods: Exosomal and plasma non-coding RNA signature associated with urinary albumin excretion in hypertension.

Riffo-Campos *et. al.*

October 22, 2021

```
doBasics = TRUE
doAll = FALSE
```

The library used for the analysis were:

```
library(Rsamtools)

## Loading required package: GenomeInfoDb
## Loading required package: BiocGenerics
## Loading required package: parallel
##
## Attaching package: 'BiocGenerics'
## The following objects are masked from 'package:parallel':
##
##     clusterApply, clusterApplyLB, clusterCall, clusterEvalQ,
##     clusterExport, clusterMap, parApply, parCapply, parLapply,
##     parLapplyLB, parRapply, parSapply, parSapplyLB
## The following objects are masked from 'package:stats':
##
##     IQR, mad, sd, var, xtabs
## The following objects are masked from 'package:base':
##
##     anyDuplicated, append, as.data.frame, basename, cbind,
colnames,
##     dirname, do.call, duplicated, eval, evalq, Filter, Find,
get, grep,
##     grepl, intersect, is.unsorted, lapply, Map, mapply, match,
mget,
##     order, paste, pmax, pmax.int, pmin, pmin.int, Position,
rank,
##     rbind, Reduce, rownames, sapply, setdiff, sort, table,
tapply,
##     union, unique, unsplit, which, which.max, which.min
## Loading required package: S4Vectors
## Loading required package: stats4
##
## Attaching package: 'S4Vectors'
```

```
## The following object is masked from 'package:base':
##
##     expand.grid
## Loading required package: IRanges
## Loading required package: GenomicRanges
## Loading required package: Biostrings
## Loading required package: XVector
##
## Attaching package: 'Biostrings'
## The following object is masked from 'package:base':
##
##     strsplit
```

```r
library(GenomicFeatures)
```

```
## Loading required package: AnnotationDbi
## Loading required package: Biobase
## Welcome to Bioconductor
##
##     Vignettes contain introductory material; view with
##     'browseVignettes()'. To cite Bioconductor, see
##     'citation("Biobase")', and for packages 'citation("pkgname")'.
```

```r
library(GenomicAlignments)
```

```
## Loading required package: SummarizedExperiment
## Loading required package: DelayedArray
## Loading required package: matrixStats
##
## Attaching package: 'matrixStats'
## The following objects are masked from 'package:Biobase':
##
##     anyMissing, rowMedians
## Loading required package: BiocParallel
##
## Attaching package: 'DelayedArray'
## The following objects are masked from 'package:matrixStats':
##
##     colMaxs, colMins, colRanges, rowMaxs, rowMins, rowRanges
## The following object is masked from 'package:Biostrings':
##
##     type
## The following objects are masked from 'package:base':
##
##     aperm, apply, rowsum
```

```r
library(edgeR)
```

```
## Loading required package: limma
##
## Attaching package: 'limma'
## The following object is masked from 'package:BiocGenerics':
##
##     plotMA
```

```r
library(VennDiagram)
```

```
## Loading required package: grid
## Loading required package: futile.logger

library(org.Hs.eg.db)

##

library(ggplot2)
```

1 ncRNAs: Data preprocessing, annotation and normalization

The quality control of the raw data was done with FastQC v0.11.8. Subsequently, the data was filtered using FASTX-Toolkit v0.013, removing adapters, read and nucleotides with low quality. The alignment was make with STAR v2.7.3a, using the GRCh38.p13 as reference genome. The SAM files were converted to BAM and sorted with SAMtools v1.10. The sorted BAM files were included in R and analyzed with the Bioconductor packages GenomicFeatures, Rsamtools, GenomicAlignments and edgeR, as below:

```
gtfFile = "gencode.v32.chr_patch_hapl_scaff.annotation.gtf"
txdb = makeTxDbFromGFF(gtfFile, format="gtf")
genes = exonsBy(txdb, by="gene")

indir = getwd()
files = list.files(indir, pattern = '*.bam')

bamLst = BamFileList(files, index=character(),
                    obeyQname=TRUE)

# Counting reads
 all_small_data121 = summarizeOverlaps(features = genes,
                                      read=bamLst,
                                      mode="Union",
                                      singleEnd=TRUE,
                                      ignore.strand=TRUE,
                                      fragments=FALSE)

# save(all_small_data121,file="all_small_data121.rda")
```

```
load("all_small_data121.rda")
dim(all_small_data121)

## [1] 67008   121

countrow = assay(all_small_data121)
boxplot(countrow[,1:40], outline=T, ylab="Counts",
       xlab="samples", main="Unnormalised data")
```

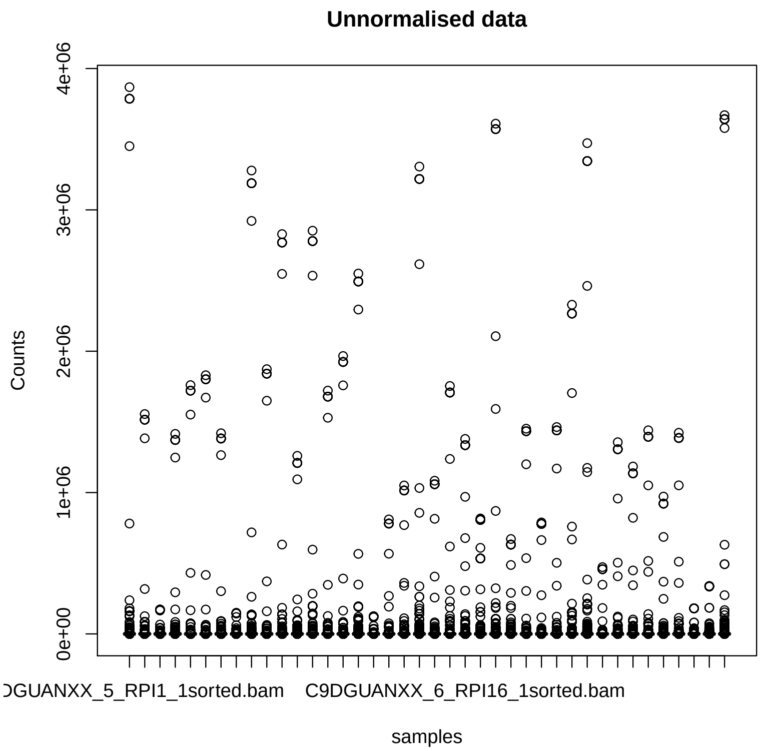


```r
## Convert to edgeR format:

edgeRmatrix_STAR = assay(all_small_data121)

metadata = read.csv(file= "metadata_small.csv",
                    header = TRUE, sep=",")

# the repeated samples were discarded by library size.
# Control_EP = 22 samples
# Control_EU = 22 samples
# Control_PP = 22 samples
# MALB_EP = 15 samples
# MALB_EU = 21 samples
# MALB_PP = 19 samples
# 121 samples

groups = factor(metadata$Group_sub,
                levels=c("control_EP","control_EU",
                         "control_PP", "MALB_EP",
                         "MALB_EU", "MALB_PP"))

STAR_DATA_y = DGEList(counts=edgeRmatrix_STAR,
                      group=groups)
dim(STAR_DATA_y)

##############################################
####### Filtering and normalization
```

```r
# Determine which genes have sufficiently large counts
# to be retained in a statistical analysis.
# genes that are not expressed in either experimental condition
keep <- filterByExpr(STAR_DATA_y)
summary(keep)
#   Mode   FALSE    TRUE
# logical   52415   14593

STAR_DATA_y_filt = STAR_DATA_y[keep, , keep.lib.sizes=FALSE]
dim(STAR_DATA_y_filt)

## annotation:

annotat = read.csv(file= "gene_ID_type.csv",
                   header = TRUE, sep=",")

STAR_Data = STAR_DATA_y_filt
rownames(STAR_Data) = annotat$x

preIDSymbol = AnnotationDbi::select(org.Hs.eg.db,
                            keys=as.character(rownames(STAR_Data)),
                            column=c("SYMBOL", "ENTREZID"),
                            keytype="ENSEMBL")

STAR_Data$genes$ENSEMBL = rownames(STAR_Data)
m = match(STAR_Data$genes$ENSEMBL, preIDSymbol$ENSEMBL)

STAR_Data$genes$SYMBOL = preIDSymbol$SYMBOL[m]
STAR_Data$genes$ENTREZID = preIDSymbol$ENTREZID[m]
STAR_Data$genes$type = as.character(annotat$gene_type)

STAR_DATA_f = STAR_Data
STAR_DATA_f$genes = as.matrix(DataFrame(STAR_Data$genes$ENSEMBL,
                                        STAR_Data$genes$SYMBOL,
                                        STAR_Data$genes$ENTREZID,
                                        STAR_Data$genes$type))

colnames(STAR_DATA_f$genes) = c("ENSEMBL","SYMBOL",
                                "ENTREZID","TYPE")
head(STAR_DATA_f$genes)
dim(STAR_DATA_f)

# Remove genes that have duplicate symbol:
d <- duplicated(STAR_DATA_f$genes[,2])
summary(d)
# Mode   FALSE    TRUE
#logical   11811    2782

#duplicates are removed and lib size is recalculated:
STAR_DATA_f = STAR_DATA_f[!d, , keep.lib.sizes=FALSE]
dim(STAR_DATA_f)

# The most used method (recommended) to normalize is:
smallRNA_STAR_itype = calcNormFactors(STAR_DATA_f)
```

```r
# save(smallRNA_STAR_itype,file="smallRNA_STAR_itype.rda")

# The table with the types of genes for each group:
gene_type_all = DataFrame(smallRNA_STAR_itype$genes)
table_RNA_Type = smallRNA_STAR_itype$counts
colnames(table_RNA_Type) = metadata$Group_sub
rownames(table_RNA_Type) = gene_type_all$TYPE

# write.csv(table_RNA_Type, file = "sample_matrix_grouptype.csv")

sum(smallRNA_STAR_itype[["samples"]][["lib.size"]])
# [1] 402974868
```

2 ncRNAs: Statistic Analysis

```r
load("smallRNA_STAR_itype.rda")
dim(smallRNA_STAR_itype)

## [1] 11811   121

metadata = read.csv(file= "metadata_small.csv",
                      header = TRUE, sep=",")

groups = factor(metadata$Group_sub,
                  levels=c("control_EP","control_EU",
                           "control_PP", "MALB_EP",
                           "MALB_EU", "MALB_PP"))
groups2 = factor(metadata$Gender,
                  levels=c("male","female"))

design = model.matrix(~0+groups+groups2)
colnames(design) = c("control_EP","control_EU",
                      "control_PP", "MALB_EP",
                      "MALB_EU", "MALB_PP", "Sex")

design[70:80,]

##    control_EP control_EU control_PP MALB_EP MALB_EU MALB_PP Sex
## 70          0          0          0       1       0       0   0
## 71          1          0          0       0       0       0   1
## 72          0          0          0       1       0       0   0
## 73          0          0          0       1       0       0   0
## 74          0          0          0       1       0       0   1
## 75          0          0          0       0       1       0   0
## 76          0          1          0       0       0       0   1
## 77          0          1          0       0       0       0   0
## 78          0          1          0       0       0       0   0
## 79          0          0          0       0       1       0   0
## 80          0          1          0       0       0       0   0

y = estimateDisp(smallRNA_STAR_itype, design,
                 robust=TRUE)
plotBCV(y)
```

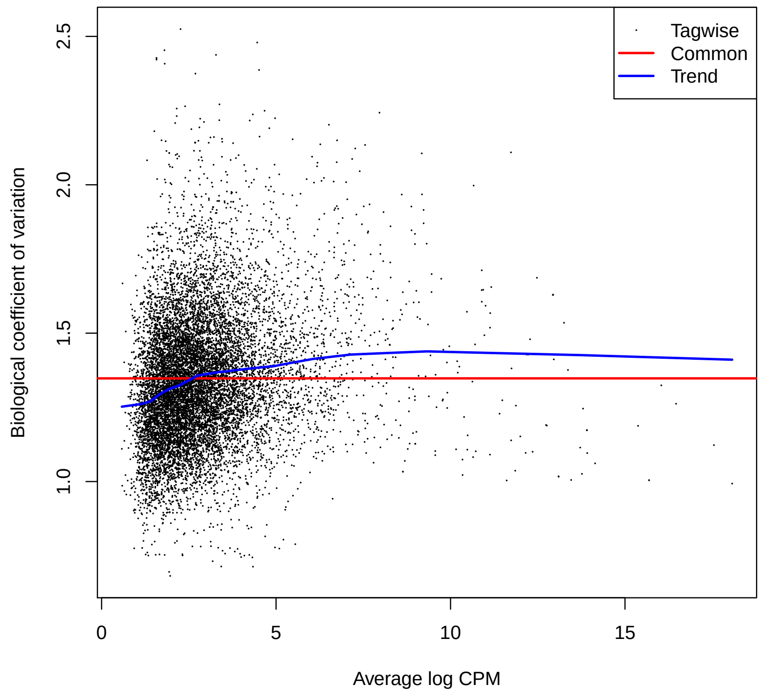


```r
fit = glmQLFit(y, design, robust=TRUE)
plotQLDisp(fit)
```

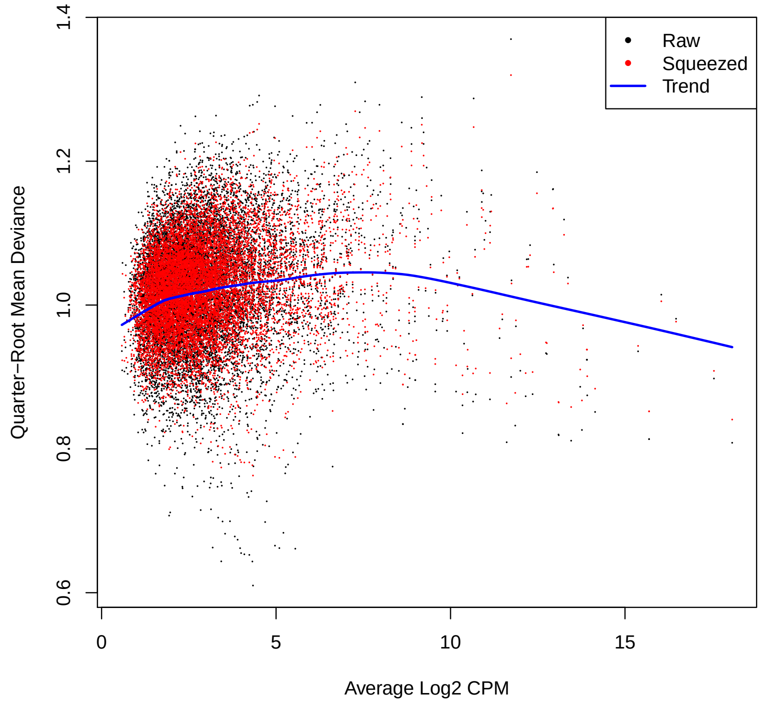

```r
my.contrasts = makeContrasts(EP=MALB_EP-control_EP,
                              EU=MALB_EU-control_EU,
                             PP=MALB_PP-control_PP,
                             levels=design)
head(my.contrasts)
```

```
##              Contrasts
## Levels        EP EU PP
##   control_EP -1  0  0
##   control_EU  0 -1  0
##   control_PP  0  0 -1
##   MALB_EP     1  0  0
##   MALB_EU     0  1  0
##   MALB_PP     0  0  1
```

```r
qlf.EP = glmQLFTest(fit, contrast= my.contrasts[,"EP"])
summary(decideTests(qlf.EP))
```

```
##        -1*control_EP 1*MALB_EP
## Down                      1893
## NotSig                    8856
## Up                        1062
```

```r
topTags(qlf.EP)
```

```
## Coefficient:  -1*control_EP 1*MALB_EP
##                         ENSEMBL  SYMBOL ENTREZID           TYPE    logFC
## ENSG00000143367 ENSG00000143367   TUFT1     7286 protein_coding 5.556850
## ENSG00000135540 ENSG00000135540   NHSL1    57224 protein_coding 4.873782
## ENSG00000005249 ENSG00000005249 PRKAR2B     5577 protein_coding 4.773597
## ENSG00000186184 ENSG00000186184  POLR1D    51082 protein_coding 5.119096
## ENSG00000062194 ENSG00000062194   GPBP1    65056 protein_coding 3.958838
## ENSG00000147246 ENSG00000147246   HTR2C     3358 protein_coding 6.346510
## ENSG00000109332 ENSG00000109332  UBE2D3     7323 protein_coding 4.882039
## ENSG00000116497 ENSG00000116497 S100PBP    64766 protein_coding 4.795788
## ENSG00000131351 ENSG00000131351   HAUS8    93323 protein_coding 6.235180
## ENSG00000166387 ENSG00000166387 PPFIBP2     8495 protein_coding 3.768385
##                   logCPM        F       PValue          FDR
## ENSG00000143367 3.733902 44.30813 5.676307e-10 3.603337e-06
## ENSG00000135540 3.200192 44.11486 6.101663e-10 3.603337e-06
## ENSG00000005249 3.687640 41.11444 1.990651e-09 6.454497e-06
## ENSG00000186184 6.915442 40.40706 2.636931e-09 6.454497e-06
## ENSG00000062194 1.650876 40.28897 2.732409e-09 6.454497e-06
## ENSG00000147246 7.040030 38.96942 4.686703e-09 8.258977e-06
## ENSG00000109332 3.759815 38.86132 4.894830e-09 8.258977e-06
## ENSG00000116497 4.363380 37.61213 8.103999e-09 1.130926e-05
## ENSG00000131351 4.757662 37.29828 9.204012e-09 1.130926e-05
## ENSG00000166387 3.654012 37.17079 9.575194e-09 1.130926e-05
```

```r
DE_EP = topTags(qlf.EP, n=10000, adjust.method="BH",
                sort.by="PValue")
# write.csv(DE_EP, file = "DE_EP_pvalSex.csv")

qlf.EU = glmQLFTest(fit, contrast= my.contrasts[,"EU"])
summary(decideTests(qlf.EU))
```

```
##        -1*control_EU 1*MALB_EU
## Down                      1434
## NotSig                    9005
## Up                        1372
```

```r
topTags(qlf.EU)
```

```
## Coefficient:  -1*control_EU 1*MALB_EU
##                         ENSEMBL  SYMBOL ENTREZID                  TYPE
## ENSG00000115365 ENSG00000115365  LANCL1    10314        protein_coding
## ENSG00000207782 ENSG00000207782  MIR150   406942                 miRNA
## ENSG00000199157 ENSG00000199157 MIR208A   406990                 miRNA
## ENSG00000112078 ENSG00000112078  KCTD20   222658        protein_coding
## ENSG00000140632 ENSG00000140632   GLYR1    84656        protein_coding
## ENSG00000215704 ENSG00000215704  CELA2B    51032        protein_coding
## ENSG00000142163 ENSG00000142163   OR1E3     8389 unprocessed_pseudogene
## ENSG00000067992 ENSG00000067992    PDK3     5165        protein_coding
## ENSG00000136982 ENSG00000136982   DSCC1    79075        protein_coding
## ENSG00000154099 ENSG00000154099  DNAAF1   123872        protein_coding
##                     logFC    logCPM        F       PValue          FDR
## ENSG00000115365  5.995436  3.621208 56.80838 5.174956e-12 6.112140e-08
## ENSG00000207782  8.194574  7.987854 50.92922 4.527267e-11 2.673577e-07
## ENSG00000199157  6.557617  2.309884 48.14661 1.295302e-10 4.137021e-07
## ENSG00000112078  6.419989 10.969755 47.94056 1.401074e-10 4.137021e-07
## ENSG00000140632  9.083978  7.512649 42.55066 1.128927e-09 2.666752e-06
## ENSG00000215704 -5.615095  4.282332 41.73457 1.557316e-09 3.065577e-06
## ENSG00000142163  6.321624  2.971378 40.26906 2.785992e-09 4.700765e-06
## ENSG00000067992  4.249798  3.597367 39.20681 4.247515e-09 6.081602e-06
## ENSG00000136982  5.434681  2.628363 38.82755 4.961744e-09 6.081602e-06
## ENSG00000154099  4.362465  2.557801 38.73541 5.149100e-09 6.081602e-06
```

```r
DE_EU = topTags(qlf.EU, n=10000, adjust.method="BH",
                sort.by="PValue")
# write.csv(DE_EU, file = "DE_EU_pvalSex.csv")


qlf.PP = glmQLFTest(fit, contrast= my.contrasts[,"PP"])
summary(decideTests(qlf.PP))
```

```
##        -1*control_PP 1*MALB_PP
## Down                        19
## NotSig                   11781
## Up                          11
```

```r
topTags(qlf.PP)
```

```
## Coefficient:  -1*control_PP 1*MALB_PP
##                         ENSEMBL       SYMBOL  ENTREZID
## ENSG00000177272 ENSG00000177272        KCNA3      3738
## ENSG00000159256 ENSG00000159256        MORC3     23515
## ENSG00000106628 ENSG00000106628        POLD2      5425
## ENSG00000119689 ENSG00000119689         DLST      1743
## ENSG00000132825 ENSG00000132825      PPP1R3D      5509
## ENSG00000217801 ENSG00000217801 LOC100288175 100288175
## ENSG00000183864 ENSG00000183864         TOB2     10766
```

```
## ENSG00000205683 ENSG00000205683          DPF3      8110
## ENSG00000100241 ENSG00000100241          SBF1      6305
## ENSG00000106609 ENSG00000106609       TMEM248     55069
##                                                    TYPE     logFC   logCPM        F
## ENSG00000177272                          protein_coding  7.701729 6.788017 48.87019
## ENSG00000159256                          protein_coding  4.939211 3.733923 33.93634
## ENSG00000106628                          protein_coding -6.033276 3.571365 29.75033
## ENSG00000119689                          protein_coding -5.440633 3.934173 29.60532
## ENSG00000132825                          protein_coding -4.494360 7.764530 27.92190
## ENSG00000217801 transcribed_unprocessed_pseudogene -5.270231 2.929376 26.56934
## ENSG00000183864                          protein_coding -4.779342 3.003335 25.70465
## ENSG00000205683                          protein_coding  3.810950 3.480115 24.70420
## ENSG00000100241                          protein_coding  3.556271 4.557625 23.95785
## ENSG00000106609                          protein_coding -3.974551 2.586897 23.10181
##                       PValue          FDR
## ENSG00000177272 9.839409e-11 1.162133e-06
## ENSG00000159256 3.655901e-08 2.158993e-04
## ENSG00000106628 2.124590e-07 6.673609e-04
## ENSG00000119689 2.260133e-07 6.673609e-04
## ENSG00000132825 4.654971e-07 1.099597e-03
## ENSG00000217801 8.369635e-07 1.647563e-03
## ENSG00000183864 1.221465e-06 2.060960e-03
## ENSG00000205683 1.897222e-06 2.801012e-03
## ENSG00000100241 2.640617e-06 3.465370e-03
## ENSG00000106609 3.867083e-06 4.567411e-03

DE_PP = topTags(qlf.PP, n=10000, adjust.method="BH",
                sort.by="PValue")
  # write.csv(DE_PP, file = "DE_PP_pvalSex.csv")
```

3 piRNAs: Data preprocessing, annotation and normalization

The quality control of the raw data was done with FastQC v0.11.8. Subsequently, the data was filtered using FASTX-Toolkit v0.013, removing adapters, read and nucleotides with low quality. The alignment was make with STAR v2.7.3a, using the GRCh38.p13 as reference genome. The SAM files were converted to BAM and sorted with SAMtools v1.10. The sorted BAM files were included in R and analyzed with the bioconductor packages GenomicFeatures, Rsamtools, GenomicAlignments and edgeR, as below:

```
  getwd()
  gtfFile = "modified_pirnadb_v1_7_6_hg38.gtf"
  txdb = makeTxDbFromGFF(gtfFile, format="gtf")
  genes = transcriptsBy(txdb, by="gene")

  indir = getwd()
  files = list.files(indir, pattern = '*.bam')

  bamLst = BamFileList(files, index=character(),
                   obeyQname=TRUE)
```

```r
# Counting reads
piRNA_data121 = summarizeOverlaps(features = genes,
                                  read=bamLst,
                                  mode="Union",
                                  singleEnd=TRUE,
                                  ignore.strand=TRUE,
                                  fragments=FALSE)

# save(piRNA_data121,file="piRNA_data121.rda")
```

```r
load("piRNA_data121.rda")
dim(piRNA_data121)
```

```
## [1] 812384    121
```

```r
countrow = assay(piRNA_data121)
boxplot(countrow, outline=T, ylab="Counts",
    xlab="samples", main="Unnormalised data")
```

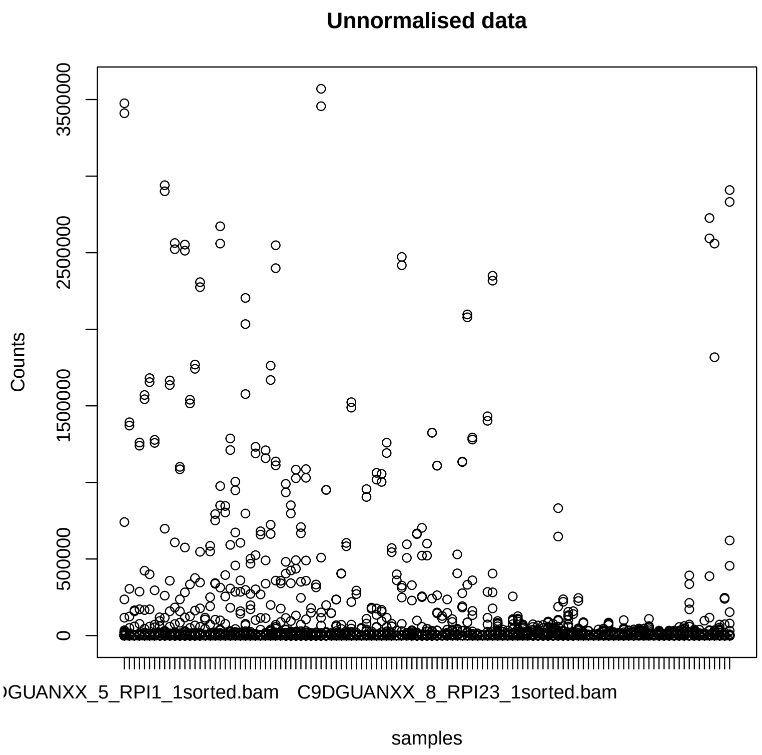


```r
## Convert to edgeR format:

edgeRmatrix_STAR = assay(piRNA_data121)

metadata = read.csv(file= "metadata_small.csv",
                    header = TRUE, sep=",")

  # the repeated samples were descarted by library size.
```

```r
  # Control_EP = 22 samples
  # Control_EU = 22 samples
  # Control_PP = 22 samples
  # MALB_EP = 15 samples
  # MALB_EU = 21 samples
  # MALB_PP = 19 samples
  # 121 samples

groups = factor(metadata$Group_sub,
                 levels=c("control_EP","control_EU",
                          "control_PP", "MALB_EP",
                          "MALB_EU", "MALB_PP"))

STAR_DATA_y = DGEList(counts=edgeRmatrix_STAR,
                      group=groups)
dim(STAR_DATA_y)

  ##############################################
  ####### Filtering and normalization

  # Determine which genes have sufficiently large counts
  # to be retained in a statistical analysis.
  # genes that are not expressed in either experimental condition
keep <- filterByExpr(STAR_DATA_y)
summary(keep)
  #   Mode   FALSE    TRUE
  #logical  812091     293

STAR_DATA_y_filt = STAR_DATA_y[keep, , keep.lib.sizes=FALSE]
dim(STAR_DATA_y_filt)


  # The most used method (recommended) to normalize is:
piRNA_STAR_itype = calcNormFactors(STAR_DATA_y_filt)

  # save(piRNA_STAR_itype,file="piRNA_STAR_itype.rda")

# The table with the types of piRNA genes for each group:
table_RNA_Type = piRNA_STAR_itype$counts
colnames(table_RNA_Type) = metadata$Group_sub

# write.csv(table_RNA_Type, file = "Matrix_count_norm_anot_piRNA.csv")

sum(piRNA_STAR_itype[["samples"]][["lib.size"]])
# [1] 244217613
```

4 piRNAs: Statistic Analysis

```r
load("piRNA_STAR_itype.rda")
dim(piRNA_STAR_itype)

## [1] 293 121
```

```r
metadata = read.csv(file= "metadata_small.csv",
                    header = TRUE, sep=",")

groups = factor(metadata$Group_sub,
                  levels=c("control_EP","control_EU",
                           "control_PP", "MALB_EP",
                           "MALB_EU", "MALB_PP"))
groups2 = factor(metadata$Gender,
                  levels=c("male","female"))

design = model.matrix(~0+groups+groups2)
colnames(design) = c("control_EP","control_EU",
                     "control_PP", "MALB_EP",
                     "MALB_EU", "MALB_PP", "Sex")
design[70:80,]
```

```
##    control_EP control_EU control_PP MALB_EP MALB_EU MALB_PP Sex
## 70          0          0          0       1       0       0   0
## 71          1          0          0       0       0       0   1
## 72          0          0          0       1       0       0   0
## 73          0          0          0       1       0       0   0
## 74          0          0          0       1       0       0   1
## 75          0          0          0       0       1       0   0
## 76          0          1          0       0       0       0   1
## 77          0          1          0       0       0       0   0
## 78          0          1          0       0       0       0   0
## 79          0          0          0       0       1       0   0
## 80          0          1          0       0       0       0   0
```

```r
y <- estimateDisp(piRNA_STAR_itype, design, robust=TRUE)
y$common.dispersion
```

```
## [1] 2.779125
```

```r
plotBCV(y)
```

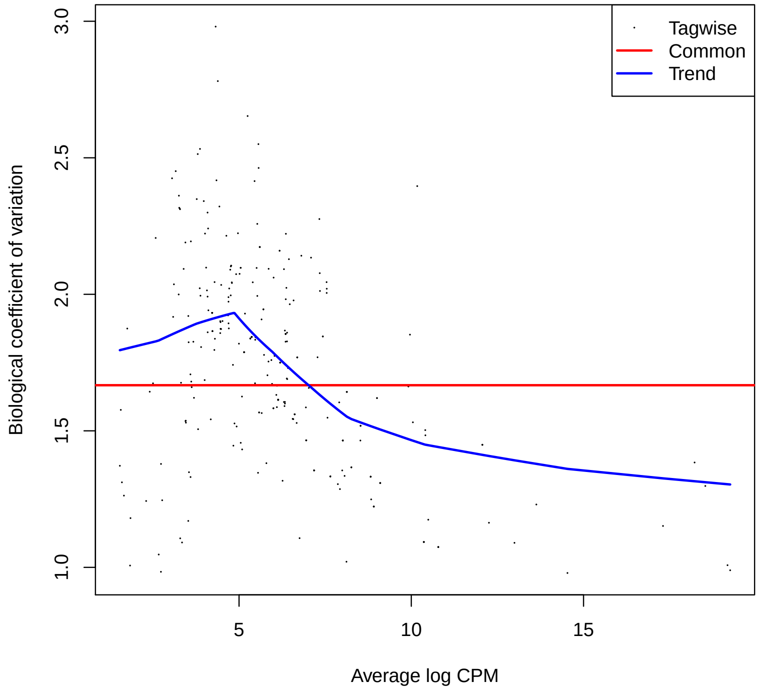


```
fit <- glmQLFit(y, design, robust=TRUE)
plotQLDisp(fit)
```

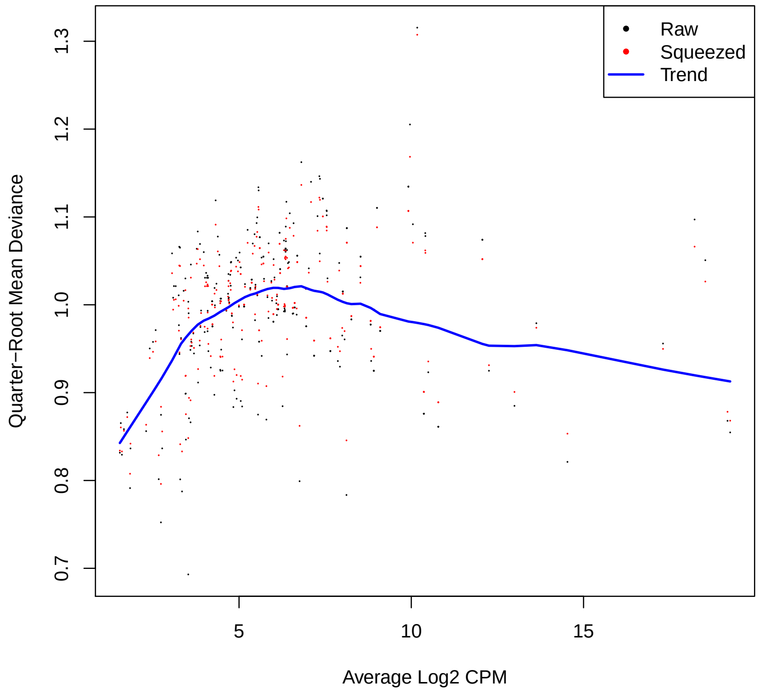

```
my.contrasts = makeContrasts(EP=MALB_EP-control_EP,
                             EU=MALB_EU-control_EU,
                             PP=MALB_PP-control_PP,
                             levels=design)

head(my.contrasts)

##              Contrasts
## Levels        EP EU PP
##   control_EP -1  0  0
##   control_EU  0 -1  0
##   control_PP  0  0 -1
##   MALB_EP     1  0  0
##   MALB_EU     0  1  0
##   MALB_PP     0  0  1

qlf.EP = glmQLFTest(fit, contrast= my.contrasts[,"EP"])
summary(decideTests(qlf.EP))

##        -1*control_EP 1*MALB_EP
## Down                        24
## NotSig                     251
## Up                          18

topTags(qlf.EP)

## Coefficient:  -1*control_EP 1*MALB_EP
##                          logFC   logCPM        F       PValue          FDR
## hsa-piR-32989         5.449725 4.793614 24.01367 2.529341e-06 0.0003941425
## hsa-piR-32989-722978  5.438756 4.790075 23.87513 2.690393e-06 0.0003941425
## hsa-piR-33056         5.030508 7.331912 19.61460 1.854445e-05 0.0018111746
## hsa-piR-1613-170729   4.647298 6.177259 17.89808 4.112948e-05 0.0024116499
## hsa-piR-1613-170730   4.647149 6.177122 17.89678 4.115444e-05 0.0024116499
## hsa-piR-28595-609487 -4.233630 2.580194 16.67224 7.317078e-05 0.0032525925
## hsa-piR-33043        -3.844711 1.568218 16.68333 7.770699e-05 0.0032525925
## hsa-piR-27306        -3.586696 7.995429 15.64958 1.188978e-04 0.0043546310
## hsa-piR-32186-672265 -4.530117 7.544610 15.18255 1.486365e-04 0.0048389439
## hsa-piR-32186-672267 -4.414666 7.546293 14.30961 2.262141e-04 0.0066280730

DE_EP = topTags(qlf.EP, n=1000, adjust.method="BH",
                sort.by="PValue")
# write.csv(DE_EP, file = "DE_EP_sex.csv")


qlf.EU = glmQLFTest(fit, contrast= my.contrasts[,"EU"])
summary(decideTests(qlf.EU))

##        -1*control_EU 1*MALB_EU
## Down                        26
## NotSig                     246
## Up                          21

topTags(qlf.EU)

## Coefficient:  -1*control_EU 1*MALB_EU
##                          logFC    logCPM        F       PValue          FDR
```

```
## hsa-piR-1961         -6.013636  4.227959 32.97183 5.291561e-08 3.876068e-06
## hsa-piR-1961-201334  -6.013636  4.227959 32.97183 5.291561e-08 3.876068e-06
## hsa-piR-1961-201335  -6.013636  4.227959 32.97183 5.291561e-08 3.876068e-06
## hsa-piR-1961-201336  -6.013636  4.227959 32.97183 5.291561e-08 3.876068e-06
## hsa-piR-27484        -5.012692  4.823642 25.35911 1.393347e-06 8.165011e-05
## hsa-piR-20478        -5.178928  6.583289 23.00530 3.969792e-06 1.938582e-04
## hsa-piR-33009         3.841519  3.692113 20.02586 1.534818e-05 6.113820e-04
## hsa-piR-33169         3.638423  4.482558 20.07747 1.669302e-05 6.113820e-04
## hsa-piR-33013-723196  2.251976 17.307161 16.73650 7.098333e-05 2.310902e-03
## hsa-piR-28340        -3.464037  3.392198 13.60750 3.179593e-04 9.316207e-03
```

```
DE_EU = topTags(qlf.EU, n=1000, adjust.method="BH",
                sort.by="PValue")
# write.csv(DE_EU, file = "DE_EU_sex.csv")


qlf.PP = glmQLFTest(fit, contrast= my.contrasts[,"PP"])
summary(decideTests(qlf.PP))
```

```
##        -1*control_PP 1*MALB_PP
## Down                         0
## NotSig                     292
## Up                           1
```

```
topTags(qlf.PP)
```

```
## Coefficient:  -1*control_PP 1*MALB_PP
##                          logFC    logCPM         F       PValue        FDR
## hsa-piR-32853         3.255512 10.046461 15.648492 0.0001189593 0.03485506
## hsa-piR-32936        -2.830912  3.588810 10.674192 0.0013564473 0.17811997
## hsa-piR-23399        -3.415084  5.398523 10.088030 0.0018237540 0.17811997
## hsa-piR-32987-722937  2.191191  2.408007  8.250136 0.0046872430 0.26974149
## hsa-piR-33169         2.385953  4.482558  8.059776 0.0052909455 0.26974149
## hsa-piR-786           2.100516  6.395131  7.648696 0.0064212872 0.26974149
## hsa-piR-28739         2.460957  4.632513  7.416117 0.0073976028 0.26974149
## hsa-piR-33049         2.279245  7.342823  7.205656 0.0081141461 0.26974149
## hsa-piR-1475          2.325696  3.879952  7.230169 0.0082855748 0.26974149
## hsa-piR-33164         2.447063  6.359981  6.889957 0.0095977128 0.28121298
```

```
DE_PP = topTags(qlf.PP, n=1000, adjust.method="BH",
                sort.by="PValue")
# write.csv(DE_PP, file = "DE_PP_sex.csv")
```

4.1 Figure 1

```
percent= read.csv(file= "Figure2.csv",
                  header = TRUE, sep=",")

#--------------------------------------------------
dfCpp = data.frame("brand" = percent$Type,
            "share" = percent$Control.PP.total)

# Create a basic bar
```

```r
pie = ggplot(dfCpp, aes(x="", y=share, fill=brand)) +
  geom_bar(stat="identity", width=1, color = "white")

# Convert to pie (polar coordinates) and add labels
pie = pie + coord_polar("y", start=0)

# Add color scale (hex colors)
pie = pie + scale_fill_manual(values=c("#a31c15", "#fac505",
                                       "#49e6e3", "#ae75eb",
                                       "#04bf42", "#227067"),
                              breaks = waiver())

# Remove labels and add title
pie = pie + labs(x = NULL, y = NULL, fill = NULL, title = NULL)

# Tidy up the theme
pie = pie + theme_classic() + theme(axis.line = element_blank(),
          axis.text = element_blank(),
          axis.ticks = element_blank())
pie
```

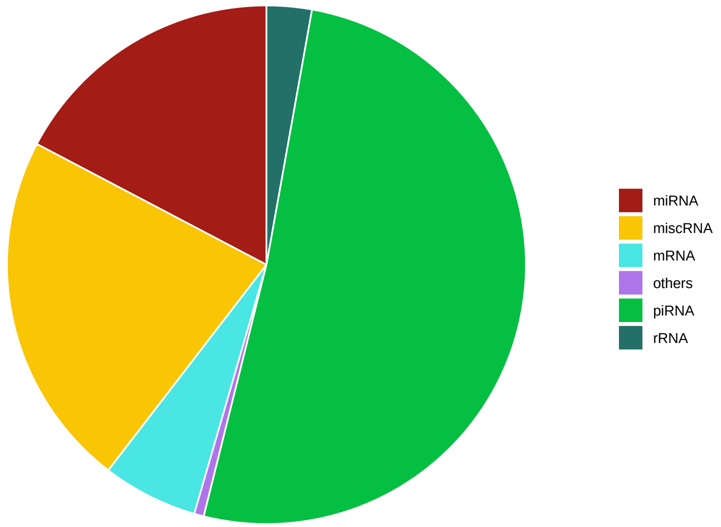


```r
#------------------------------------------------
dfMpp = data.frame("brand" = percent$Type,
            "share" = percent$MALB.PP.total)

pie = ggplot(dfMpp, aes(x="", y=share, fill=brand)) +
  geom_bar(stat="identity", width=1, color = "white")
pie = pie + coord_polar("y", start=0)
```

```
pie = pie + scale_fill_manual(values=c("#a31c15", "#fac505",
                                       "#49e6e3", "#ae75eb",
                                       "#04bf42", "#227067"),
                              breaks = waiver())
pie = pie + labs(x = NULL, y = NULL, fill = NULL, title = NULL)
pie = pie + theme_classic() + theme(axis.line = element_blank(),
          axis.text = element_blank(),
          axis.ticks = element_blank())
pie
```

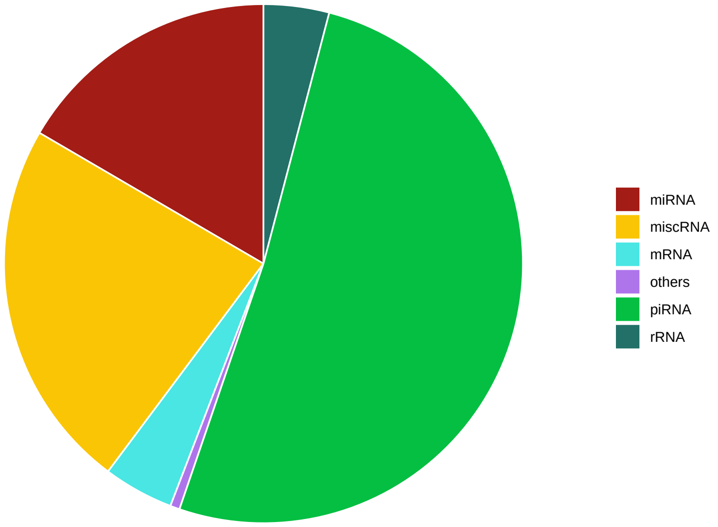


```
#--------------------------------------------------

dfCep = data.frame("brand" = percent$Type,
            "share" = percent$Control.EP.total)
pie = ggplot(dfCep, aes(x="", y=share, fill=brand)) +
  geom_bar(stat="identity", width=1, color = "white")
pie = pie + coord_polar("y", start=0)
pie = pie + scale_fill_manual(values=c("#a31c15", "#fac505",
                                       "#49e6e3", "#ae75eb",
                                       "#04bf42", "#227067"),
                              breaks = waiver())
pie = pie + labs(x = NULL, y = NULL, fill = NULL, title = NULL)
pie = pie + theme_classic() + theme(axis.line = element_blank(),
          axis.text = element_blank(),
          axis.ticks = element_blank())
pie
```

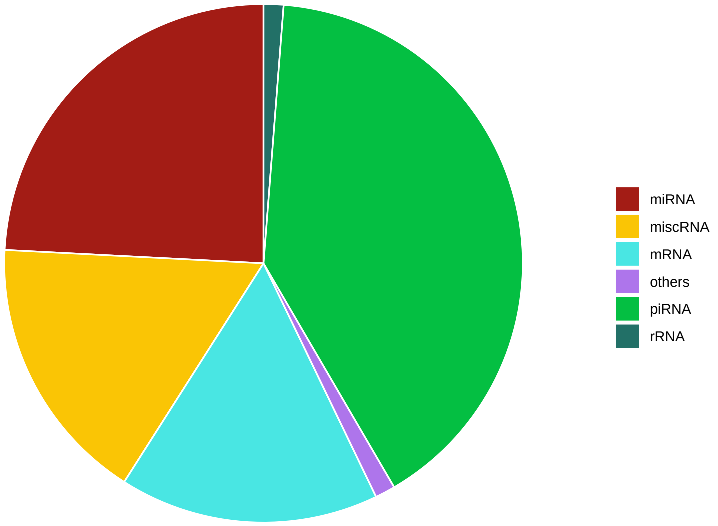


```
#-------------------------------------------------

dfMep = data.frame("brand" = percent$Type,
             "share" = percent$MALB.EP.total)

pie = ggplot(dfMep, aes(x="", y=share, fill=brand)) +
  geom_bar(stat="identity", width=1, color = "white")
pie = pie + coord_polar("y", start=0)
pie = pie + scale_fill_manual(values=c("#a31c15", "#fac505",
                                       "#49e6e3", "#ae75eb",
                                       "#04bf42", "#227067"),
                              breaks = waiver())
pie = pie + labs(x = NULL, y = NULL, fill = NULL, title = NULL)
pie = pie + theme_classic() + theme(axis.line = element_blank(),
          axis.text = element_blank(),
          axis.ticks = element_blank())
pie
```

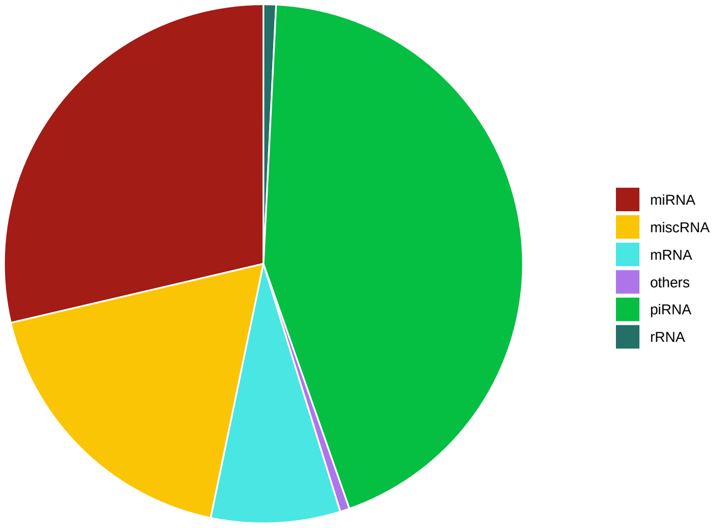


```r
#-------------------------------------------------



dfCeu = data.frame("brand" = percent$Type,
            "share" = percent$Control.EU.total)

pie = ggplot(dfCeu, aes(x="", y=share, fill=brand)) +
  geom_bar(stat="identity", width=1, color = "white")
pie = pie + coord_polar("y", start=0)
pie = pie + scale_fill_manual(values=c("#a31c15", "#fac505",
                                       "#49e6e3", "#ae75eb",
                                       "#04bf42", "#227067"),
                              breaks = waiver())
pie = pie + labs(x = NULL, y = NULL, fill = NULL, title = NULL)
pie = pie + theme_classic() + theme(axis.line = element_blank(),
          axis.text = element_blank(),
          axis.ticks = element_blank())
pie
```

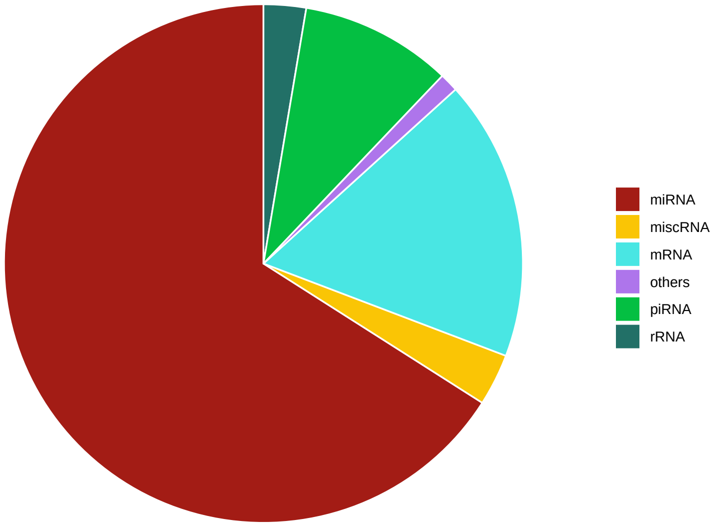


```
#-------------------------------------------------

dfMeu = data.frame("brand" = percent$Type,
            "share" = percent$MALB.EU.total)

pie = ggplot(dfMeu, aes(x="", y=share, fill=brand)) +
  geom_bar(stat="identity", width=1, color = "white")
pie = pie + coord_polar("y", start=0)
pie = pie + scale_fill_manual(values=c("#a31c15", "#fac505",
                                       "#49e6e3", "#ae75eb",
                                       "#04bf42", "#227067"),
                              breaks = waiver())
pie = pie + labs(x = NULL, y = NULL, fill = NULL, title = NULL)
pie = pie + theme_classic() + theme(axis.line = element_blank(),
          axis.text = element_blank(),
          axis.ticks = element_blank())
pie
```

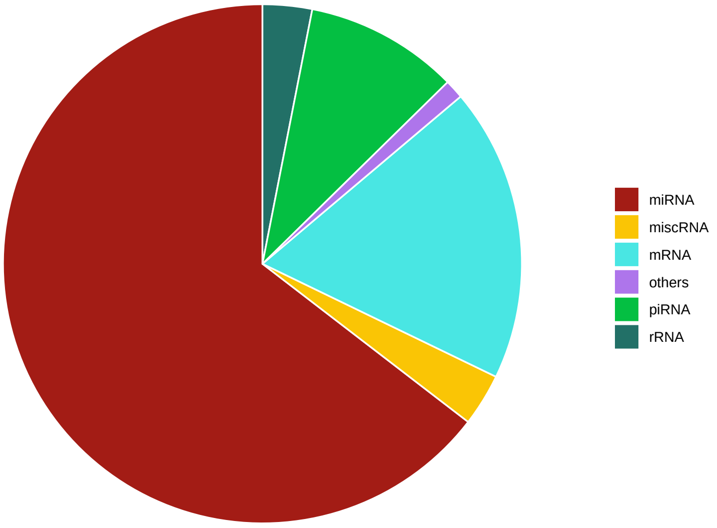


```
#--------------------------------------------------
```

4.2 Figure 2

```
diff_df_EU = read.table("DE_EU_pvalSex_all.csv",
                        sep=",", header=TRUE)
attach(diff_df_EU)
diff_df_EU["group"] <- "NotSignificant"
diff_df_EU[which(diff_df_EU['FDR'] < 0.05 &
                   abs(diff_df_EU['LOG.FC']) < 2 ),
                  "group"] <- "Significant"
diff_df_EU[which(diff_df_EU['FDR'] > 0.05 &
                   abs(diff_df_EU['LOG.FC']) > 2 ),
                  "group"] <- "FoldChange"
diff_df_EU[which(diff_df_EU['FDR'] < 0.05 &
                   abs(diff_df_EU['LOG.FC']) > 2 ),
                  "group"] <- "Significant&FoldChange"


coldif = factor(diff_df_EU$group)
cols = c("#c4962b", "#333634", "#0594fa", "#1fdb44")
gpp = ggplot(data=diff_df_EU,
             aes(x=LOG.FC, y =-log10(FDR),
             colour=coldif)) +
  xlim(c(-5, 5)) +
  theme_bw() +
```

```
  theme(legend.position="none")+
  ggtitle(label = "Volcano Plot EU") +
  geom_point(color = "black", size = 0.7) +
  geom_point(size = 0.7)
gpp +
  scale_colour_manual(values = cols)+
  theme_bw(base_size = 14) +
  theme(legend.position = "right") +
  xlab(expression(log[2]("Fold Change"))) +
  ylab(expression(-log[10]("FDR"))) + # Change Y-Axis label
  geom_hline(yintercept = 1.3, colour="#990000", linetype="dashed") +
  geom_vline(xintercept = 2, colour="#990000", linetype="dashed") +
  geom_vline(xintercept = -2, colour="#990000", linetype="dashed") +
  scale_y_continuous(trans = "log1p")

## Warning: Removed 71 rows containing missing values (geom_point).
## Warning: Removed 71 rows containing missing values (geom_point).
```

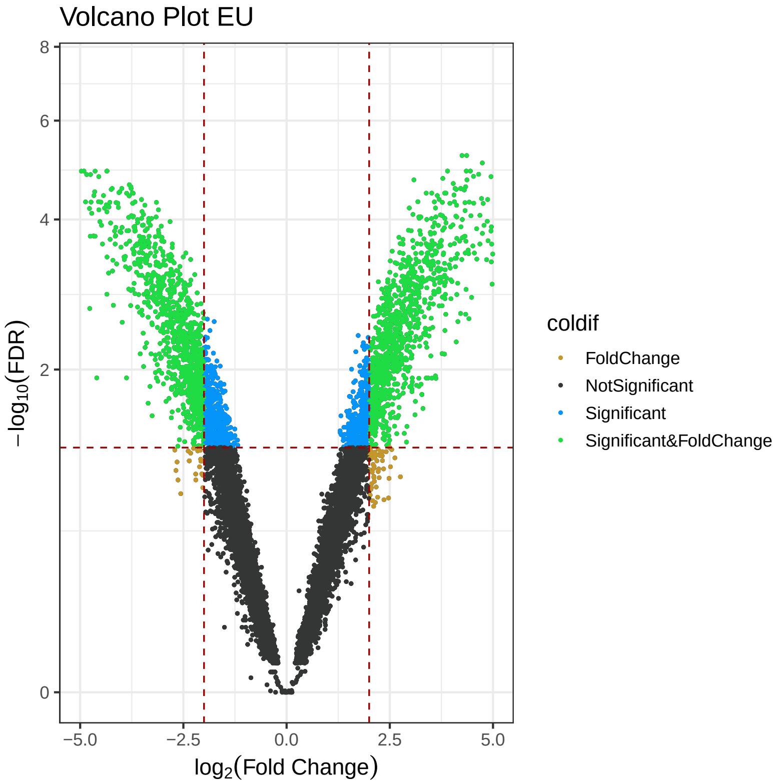


```
###-----------------------------------------------------------------
diff_df_EP <- read.table("DE_EP_pvalSex_all.csv", sep=",", header=TRUE)
attach(diff_df_EP)

## The following objects are masked from diff_df_EU:
##
##     FDR, LOG.FC, P.VALUE

diff_df_EP["group"] <- "NotSignificant"
diff_df_EP[which(diff_df_EP['FDR'] < 0.05 &
```

```
                   abs(diff_df_EP['LOG.FC']) < 2 ),
                   "group"] <- "Significant"
diff_df_EP[which(diff_df_EP['FDR'] > 0.05 &
                   abs(diff_df_EP['LOG.FC']) > 2 ),
                   "group"] <- "FoldChange"
diff_df_EP[which(diff_df_EP['FDR'] < 0.05 &
                   abs(diff_df_EP['LOG.FC']) > 2 ),
                   "group"] <- "Significant&FoldChange"


coldif = factor(diff_df_EP$group)
cols = c("#c4962b", "#333634", "#0594fa", "#1fdb44")
gpp = ggplot(data=diff_df_EP,
             aes(x=LOG.FC, y =-log10(FDR),
             colour=coldif)) +
  xlim(c(-5, 5)) +
  theme_bw() +
  theme(legend.position="none")+
  ggtitle(label = "Volcano Plot EP") +
  geom_point(color = "black", size = 0.7) +
  geom_point(size = 0.7)
gpp +
  scale_colour_manual(values = cols)+
  theme_bw(base_size = 14) +
  theme(legend.position = "right") +
  xlab(expression(log[2]("Fold Change"))) +
  ylab(expression(-log[10]("FDR"))) + # Change Y-Axis label
  geom_hline(yintercept = 1.3, colour="#990000", linetype="dashed") +
  geom_vline(xintercept = 2, colour="#990000", linetype="dashed") +
  geom_vline(xintercept = -2, colour="#990000", linetype="dashed") +
  scale_y_continuous(trans = "log1p")

## Warning: Removed 53 rows containing missing values (geom_point).
## Warning: Removed 53 rows containing missing values (geom_point).
```

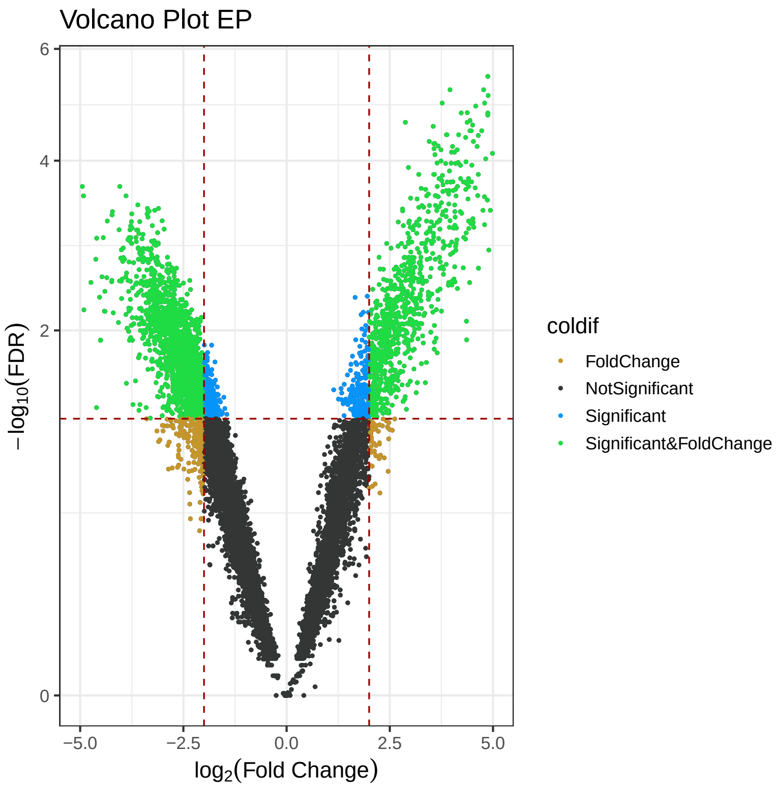


```
###-------------------------------------------------------------------

diff_df_PP <- read.table("DE_PP_pvalSex_all.csv", sep=",", header=TRUE)
attach(diff_df_PP)

## The following objects are masked from diff_df_EP:
##
##     FDR, LOG.FC, P.VALUE
##
## The following objects are masked from diff_df_EU:
##
##     FDR, LOG.FC, P.VALUE

diff_df_PP["group"] <- "NotSignificant"
diff_df_PP[which(diff_df_PP['FDR'] < 0.05 &
                   abs(diff_df_PP['LOG.FC']) < 2 ),
                   "group"] <- "Significant"
diff_df_PP[which(diff_df_PP['FDR'] > 0.05 &
                   abs(diff_df_PP['LOG.FC']) > 2 ),
                   "group"] <- "FoldChange"
diff_df_PP[which(diff_df_PP['FDR'] < 0.05 &
                   abs(diff_df_PP['LOG.FC']) > 2 ),
                   "group"] <- "Significant&FoldChange"


coldif = factor(diff_df_PP$group)
cols = c("#c4962b", "#333634", "#0594fa", "#1fdb44")
gpp = ggplot(data=diff_df_PP,
            aes(x=LOG.FC, y =-log10(FDR),
```

```
             colour=coldif)) +
  xlim(c(-5, 5)) +
  theme_bw() +
  theme(legend.position="none")+
  ggtitle(label = "Volcano Plot PP") +
  geom_point(color = "black", size = 0.7) +
  geom_point(size = 0.7)
gpp +
  scale_colour_manual(values = cols)+
  theme_bw(base_size = 14) +
  theme(legend.position = "right") +
  xlab(expression(log[2]("Fold Change"))) +
  ylab(expression(-log[10]("FDR"))) + # Change Y-Axis label
  geom_hline(yintercept = 1.3, colour="#990000", linetype="dashed") +
  geom_vline(xintercept = 2, colour="#990000", linetype="dashed") +
  geom_vline(xintercept = -2, colour="#990000", linetype="dashed") +
  scale_y_continuous(trans = "log1p")

## Warning: Removed 5 rows containing missing values (geom_point).
## Warning: Removed 5 rows containing missing values (geom_point).
```

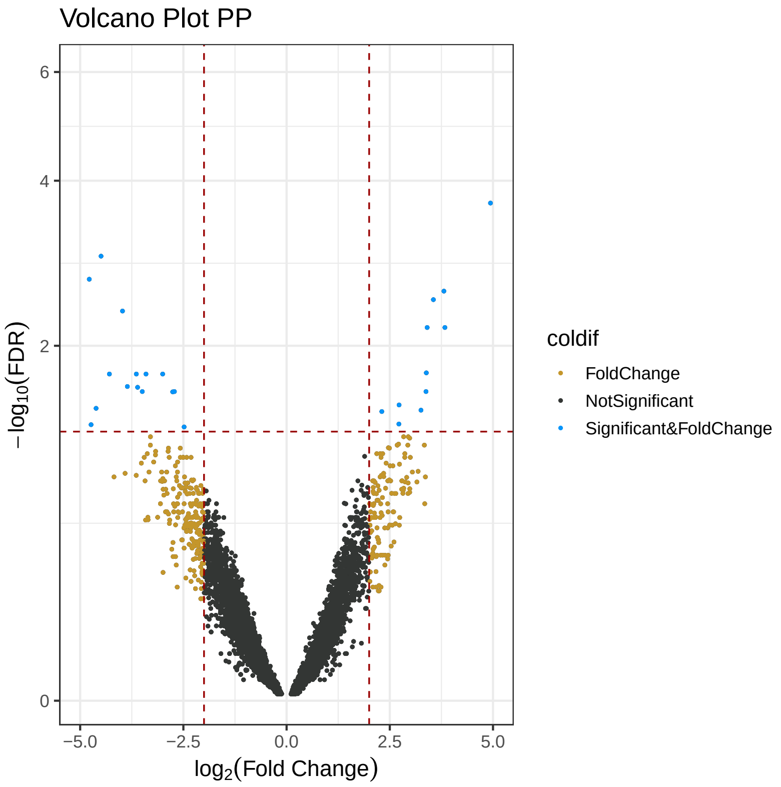

4.3 Figure 3

```
# p-val 0.05
draw.triple.venn(area1 = 483, area2 = 588, area3 = 216,
                 n12 = 185, n23 = 83, n13 = 73, n123 = 24,
                 category = c("Exosome urine", "Exosome plasma",
```

```
                              "Plasma"), lty = "blank",
                 fill = c("skyblue", "pink1", "mediumorchid"),
                 euler.d = TRUE, scaled = TRUE,
                 rotation.degree = 30, cex = 1,
                 cat.cex = 1.5, cat.dist = 0.1)
```

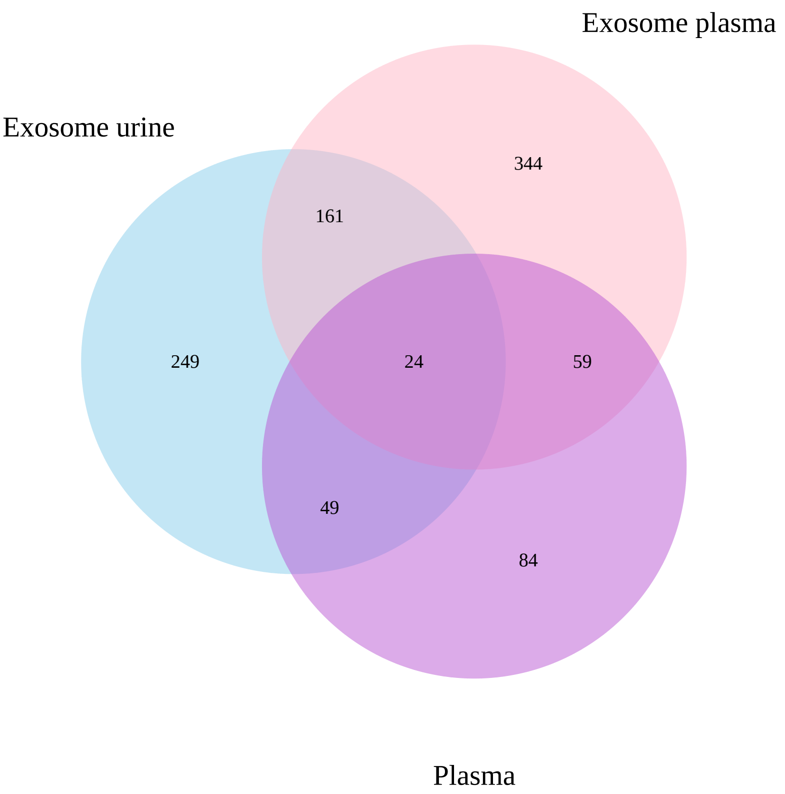


```
## (polygon[GRID.polygon.683], polygon[GRID.polygon.684], polygon[GRID.polygon.685], po

###-----------------------------------------------
########################################################

diff_df <- read.table("Common_ncRNA_Sex24.csv", sep=",", header=TRUE)
mtcars = diff_df
mtcars$ncRNA <- mtcars$ncRNA  # create new column for car names
mtcars$LogFC <- mtcars$Log_FC_PP  # compute normalized mpg
mtcars$mpg_type <- ifelse(mtcars$LogFC < 0, "Downregulated",
                          "Upregulated")  # above / below avg flag
mtcars <- mtcars[order(mtcars$LogFC), ]  # sort
mtcars$ncRNA <- factor(mtcars$ncRNA, levels = mtcars$ncRNA)
# convert to factor to retain sorted order in plot.

# Diverging Barcharts
ggplot(mtcars, aes(x=ncRNA, y=LogFC, label=LogFC)) +
  geom_bar(stat='identity', aes(fill=mpg_type), width=.5)  +
  scale_fill_manual(name="Differential expression",
                    labels = c("Upregulated", "Downregulated"),
                    values = c("Upregulated"="#00ba38",
                               "Downregulated"="#f8766d")) +
```

```r
labs(title= "Plasma") + coord_flip()
```

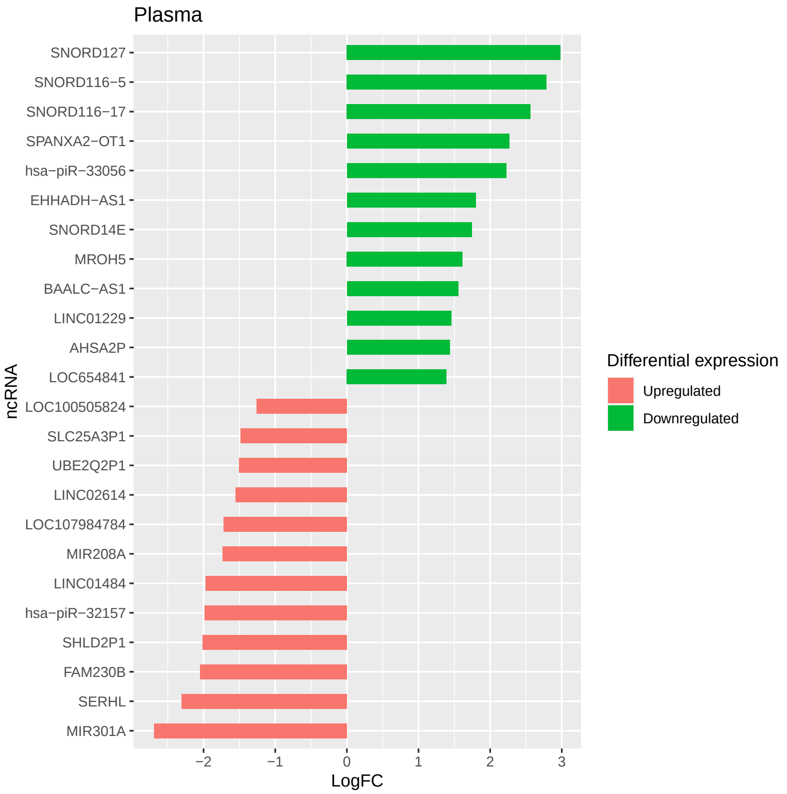


```r
#----------------------------------------------------------------
mtcars_EP = diff_df
mtcars_EP$ncRNA <- mtcars_EP$ncRNA  # create new column for car names
mtcars_EP$LogFC <- mtcars_EP$Log_FC_EP  # compute normalized mpg
mtcars_EP$mpg_type <- ifelse(mtcars_EP$LogFC < 0,
                             "Downregulated", "Upregulated")
mtcars_EP <- mtcars_EP[order(mtcars_EP$LogFC), ]  # sort
mtcars_EP$ncRNA <- factor(mtcars_EP$ncRNA,
                          levels = mtcars_EP$ncRNA)

# Diverging Barcharts
ggplot(mtcars_EP, aes(x=ncRNA, y=LogFC, label=LogFC)) +
  geom_bar(stat='identity', aes(fill=mpg_type), width=.5)  +
  scale_fill_manual(name="Differential expression",
                    labels = c("Upregulated", "Downregulated"),
                    values = c("Upregulated"="#00ba38",
                               "Downregulated"="#f8766d")) +
  labs(title= "Exosome plasma") + coord_flip()
```

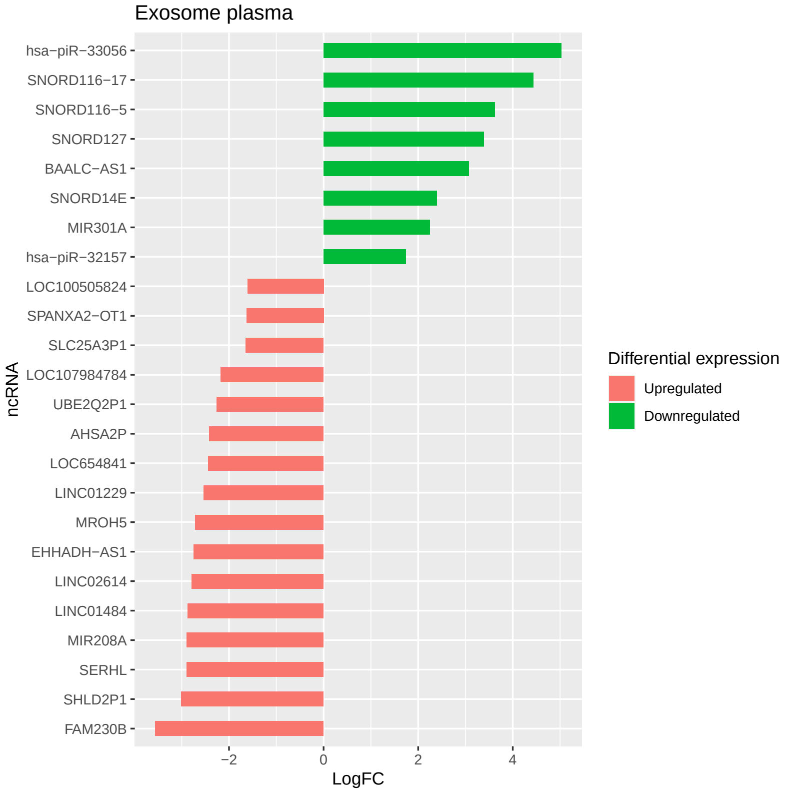


```
#----------------------------------------------
mtcars_EU = diff_df
mtcars_EU$ncRNA <- mtcars_EU$ncRNA  # create new column for car names
mtcars_EU$LogFC <- mtcars_EU$Log_FC_EU  # compute normalized mpg
mtcars_EU$mpg_type <- ifelse(mtcars_EU$LogFC < 0,
                             "Downregulated", "Upregulated")
mtcars_EU <- mtcars_EU[order(mtcars_EU$LogFC), ]  # sort
mtcars_EU$ncRNA <- factor(mtcars_EU$ncRNA, levels = mtcars_EU$ncRNA)

# Diverging Barcharts
ggplot(mtcars_EU, aes(x=ncRNA, y=LogFC, label=LogFC)) +
  geom_bar(stat='identity', aes(fill=mpg_type), width=.5)  +
  scale_fill_manual(name="Differential expression",
                    labels = c("Upregulated", "Downregulated"),
                    values = c("Upregulated"="#00ba38",
                               "Downregulated"="#f8766d")) +
  labs(title= "Exosome urine") + coord_flip()
```

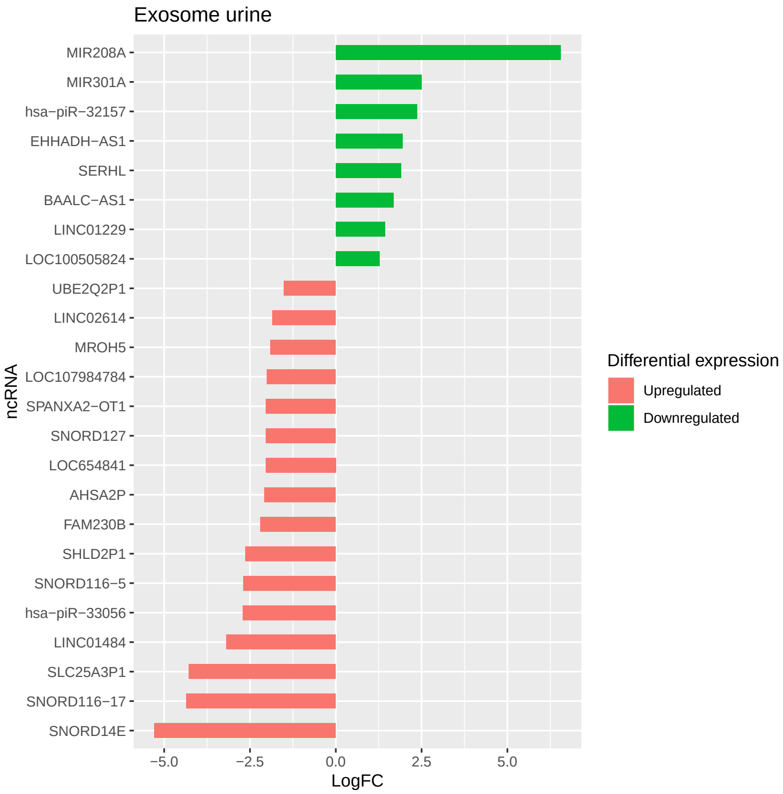

4.4 Figure 3A

```
# pval 0.05

sigtypeEP = data.frame("pubcode" = c("lncRNA","miRNA","miscRNA",
                                     "pseudogene","rRNA","scaRNA",
                                     "snoRNA","snRNA","TEC", "piRNA"),
            "percent" = c(317,137,4,65,0,2,13,1,1,48)*100/sum(317,
                                          137,4,65,0,2,13,1,1,48))

o1 = ggplot(sigtypeEP, aes(x=1, y = percent, fill = pubcode)) +
  geom_bar(stat = "identity", width=1, color = "white")
o1
```

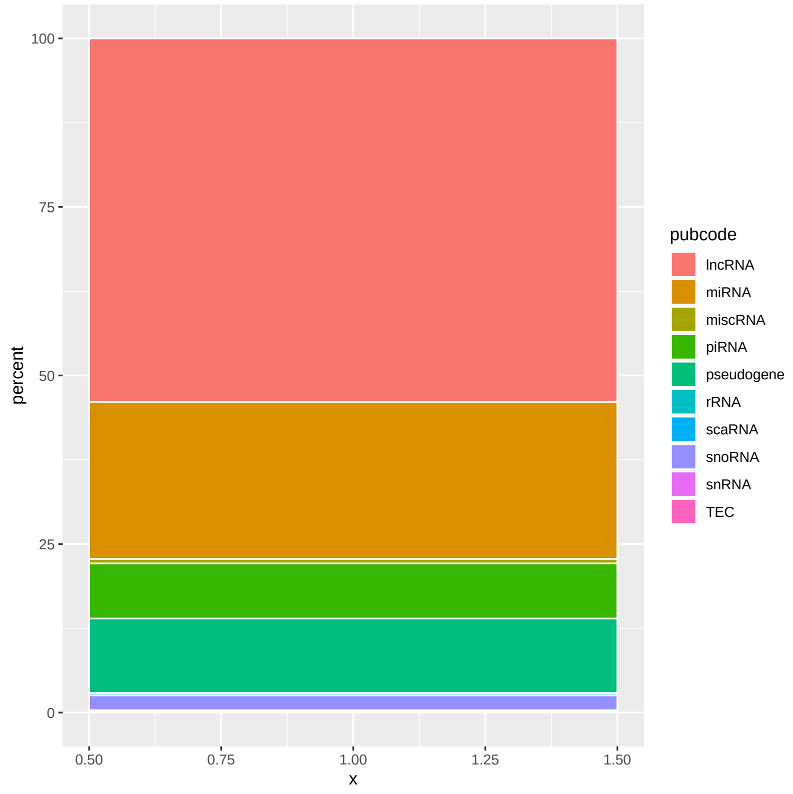


```
#-------------------------------------------------------------
sigtypeEU = data.frame("pubcode" = c("lncRNA","miRNA","miscRNA",
                                     "pseudogene","rRNA","scaRNA",
                                     "snoRNA","snRNA","TEC", "piRNA"),
            "percent" = c(269,83,1,60,0,1,19,1,0,49)*100/sum(269,
                                          83,1,60,0,1,19,1,0,49))

o1 = ggplot(sigtypeEU, aes(x=1, y = percent, fill = pubcode)) +
  geom_bar(stat = "identity", width=1, color = "white")
o1
```

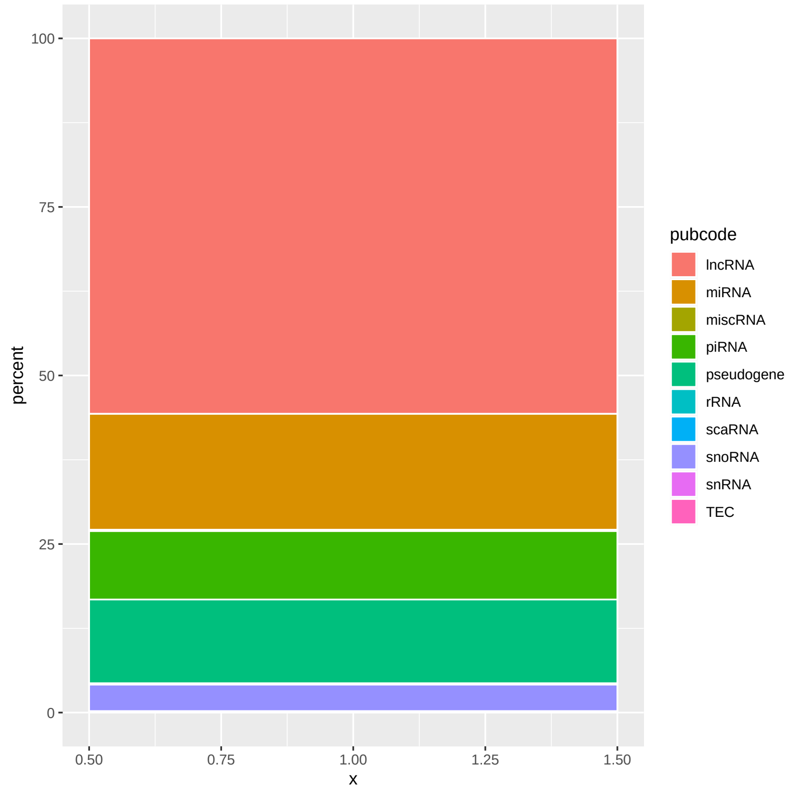


```
#-------------------------------------------------------------
sigtypePP = data.frame("pubcode" = c("lncRNA","miRNA","miscRNA",
                                     "pseudogene","rRNA","scaRNA",
                                     "snoRNA","snRNA","TEC", "piRNA"),
            "percent" = c(93,31,0,29,3,0,31,1,1,27)*100/sum(93,
                                        31,0,29,3,0,31,1,1,27))

o1 = ggplot(sigtypePP, aes(x=1, y = percent, fill = pubcode)) +
  geom_bar(stat = "identity", width=1, color = "white")
o1
```

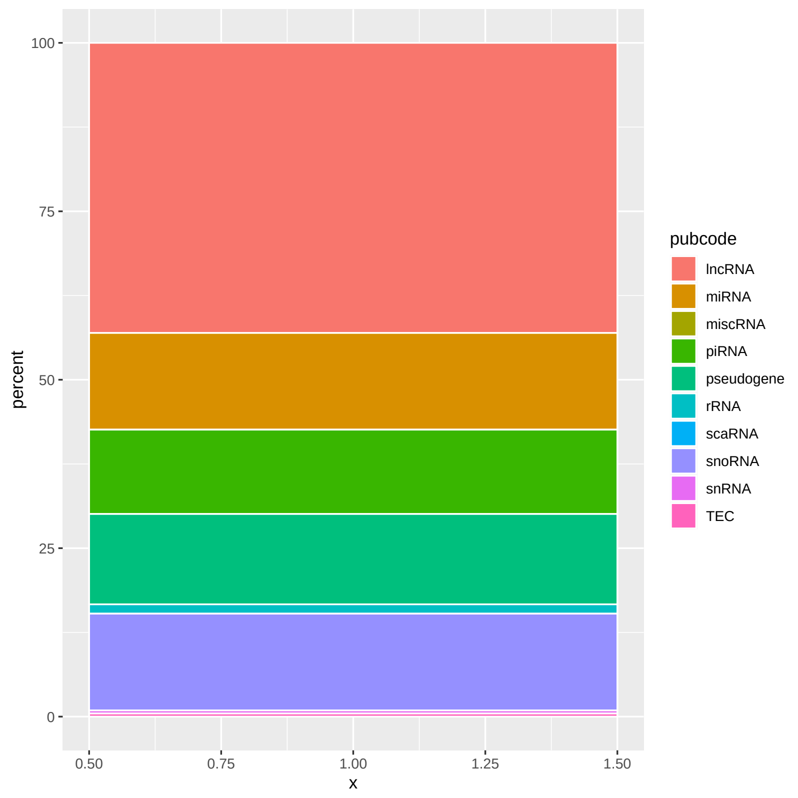

4.5 Figure supplementary 1

```
total = data.frame("brand" = c("miRNA","miscRNA","mRNA",
                               "rRNA","others", "piRNA"),
            "share" = c(32.2112093573596, 16.1830622380176, 10.3874964517704,
                        2.64487312546513, 0.838431403222684, 37.7349274241646))


pie = ggplot(total, aes(x="", y=share, fill=brand)) +
  geom_bar(stat="identity", width=1, color = "white")
pie = pie + coord_polar("y", start=0)
pie = pie + scale_fill_manual(values=c("#a31c15", "#fac505",
                                       "#49e6e3", "#ae75eb",
                                       "#04bf42", "#227067"),
                              breaks = waiver())
pie = pie + labs(x = NULL, y = NULL, fill = NULL, title = NULL)
pie = pie + theme_classic() + theme(axis.line = element_blank(),
          axis.text = element_blank(),
          axis.ticks = element_blank())

pie
```

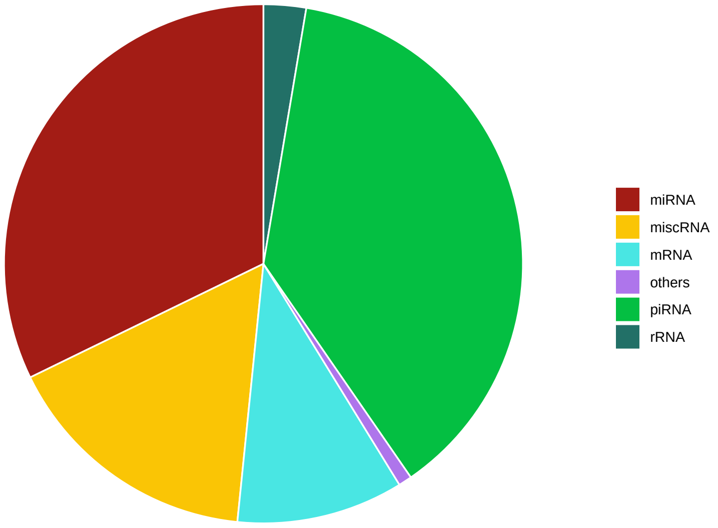


```r
total = data.frame("brand" = c("miRNA","miscRNA","mRNA",
                                "rRNA","lncRNA","pseudogene",
                                "scaRNA","snoRNA","snRNA",
                                "TEC","piRNA"),
            "share" = c(260,8,10603,4,718,159,3,43,11,2,293))

ctr <- ggplot(total, aes(x = reorder(brand,share), y = share, fill = brand)) +
  geom_bar(stat = "identity") +
  coord_flip() +
  geom_text(aes(y = share, label = share, size = 12), hjust = -0.1) +
  theme(legend.position="none") +
  labs(title=NULL, x = "RNA type", y = "n") + scale_fill_manual(values=c("#a31c15",
                              "#fac505", "#068dba", "#ae75eb", "#04bf42","#a31c15",
                              "#fac505", "#068dba", "#ae75eb", "#04bf42","#04bf12"),
                              breaks = waiver())

ctr
```

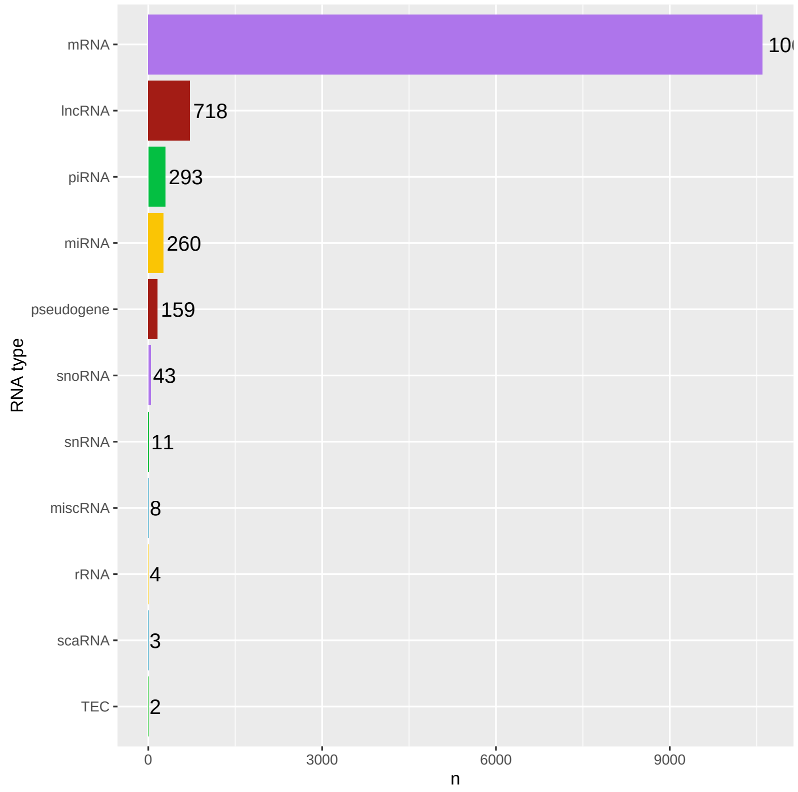

4.6 Figure supplementary 2

```
# p-val 0.05

draw.triple.venn(area1 = 4336, area2 = 4645, area3 = 1415,
                 n12 = 1667, n23 = 559, n13 = 463, n123 = 199,
                 category = c("Exosome urine", "Exosome plasma",
                              "Plasma"), lty = "blank",
                 fill = c("skyblue", "pink1", "mediumorchid"),
                 euler.d = TRUE, scaled = TRUE,
                 rotation.degree = 30, cex = 1,
                 cat.cex = 1.5, cat.dist = 0.1)
```

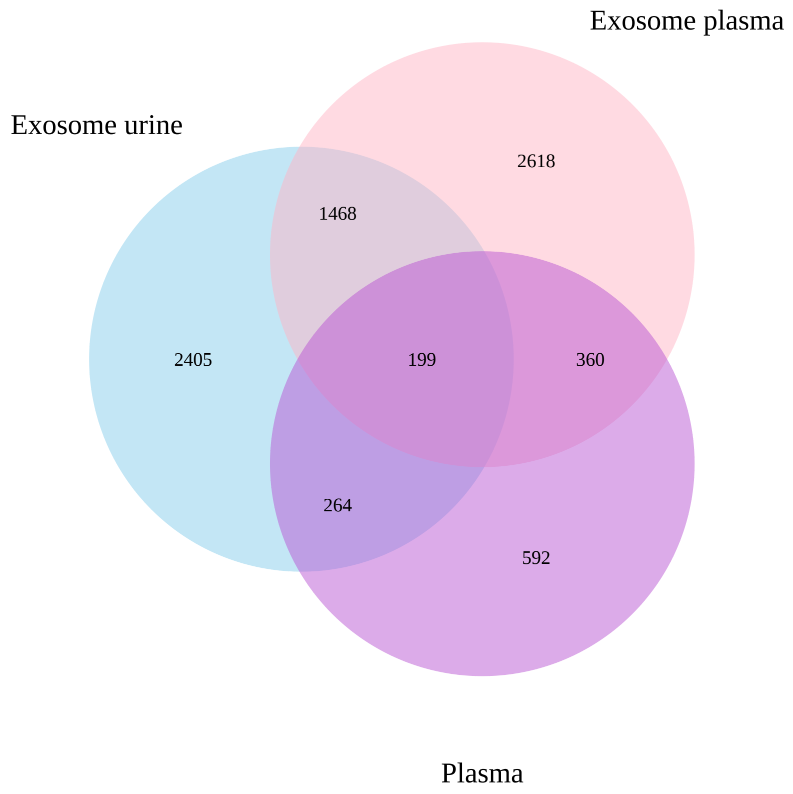


```
## (polygon[GRID.polygon.1320], polygon[GRID.polygon.1321], polygon[GRID.polygon.1322],
```

```
draw.triple.venn(area1 = 3853, area2 = 4057, area3 = 1199,
                 n12 = 1482, n23 = 476, n13 = 390, n123 = 175,
                 category = c("Exosome urine", "Exosome plasma",
                              "Plasma"), lty = "blank",
                 fill = c("skyblue", "pink1", "mediumorchid"),
                 euler.d = TRUE, scaled = TRUE,
                 rotation.degree = 30, cex = 1,
                 cat.cex = 1.5, cat.dist = 0.1)
```

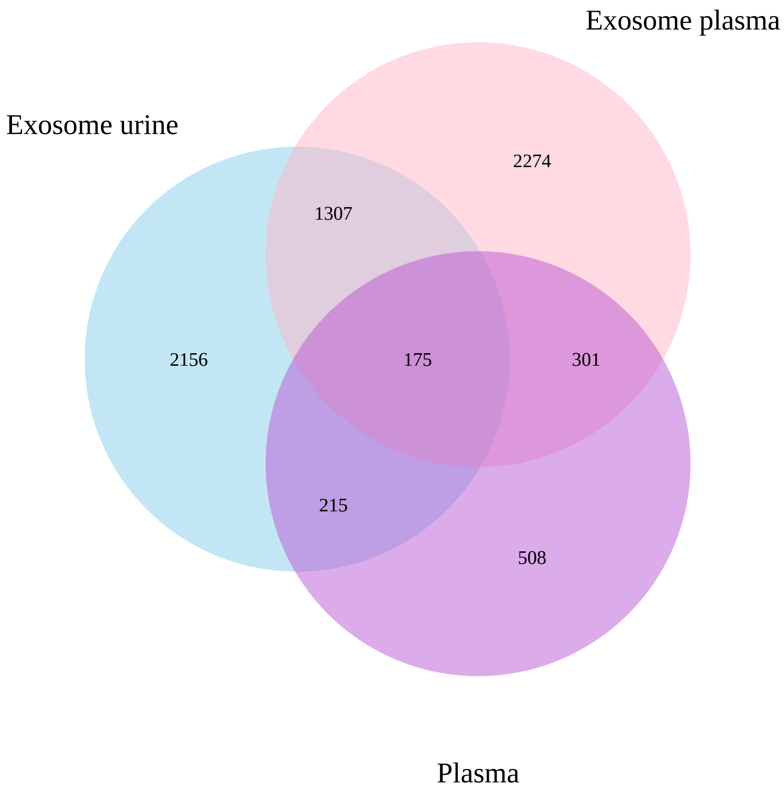


```
## (polygon[GRID.polygon.1336], polygon[GRID.polygon.1337], polygon[GRID.polygon.1338],
```

4.7 miRNAs common

```
draw.triple.venn(area1 = 11, area2 = 11, area3 = 24,
                 n12 = 0, n23 = 1, n13 = 0, n123 = 0,
                 category = c("mirtarbase", "targetscan",
                              "miRDB"), lty = "blank",
                 fill = c("skyblue", "pink1", "mediumorchid"),
                 euler.d = TRUE, scaled = TRUE,
                 rotation.degree = 30, cex = 1,
                 cat.cex = 1.5, cat.dist = 0.1)
```

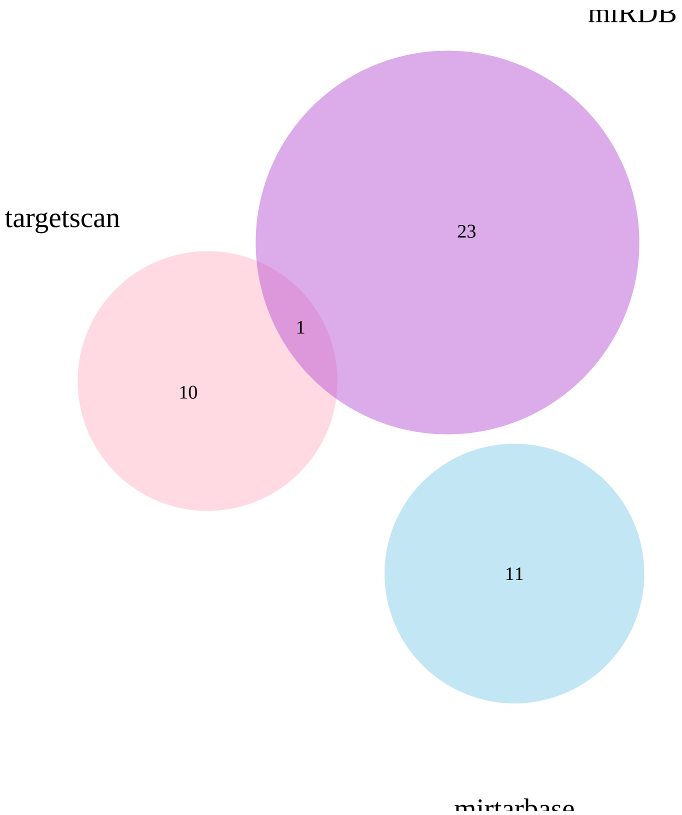


```
## (polygon[GRID.polygon.1352], polygon[GRID.polygon.1353], polygon[GRID.polygon.1354],
```

```
draw.triple.venn(area1 = 130, area2 = 18, area3 = 176,
                 n12 = 2, n23 = 5, n13 = 27, n123 = 0,
                 category = c("mirtarbase", "targetscan",
                              "miRDB"), lty = "blank",
                 fill = c("skyblue", "pink1", "mediumorchid"),
                 euler.d = TRUE, scaled = TRUE,
                 rotation.degree = 30, cex = 1,
                 cat.cex = 1.5, cat.dist = 0.1)
```

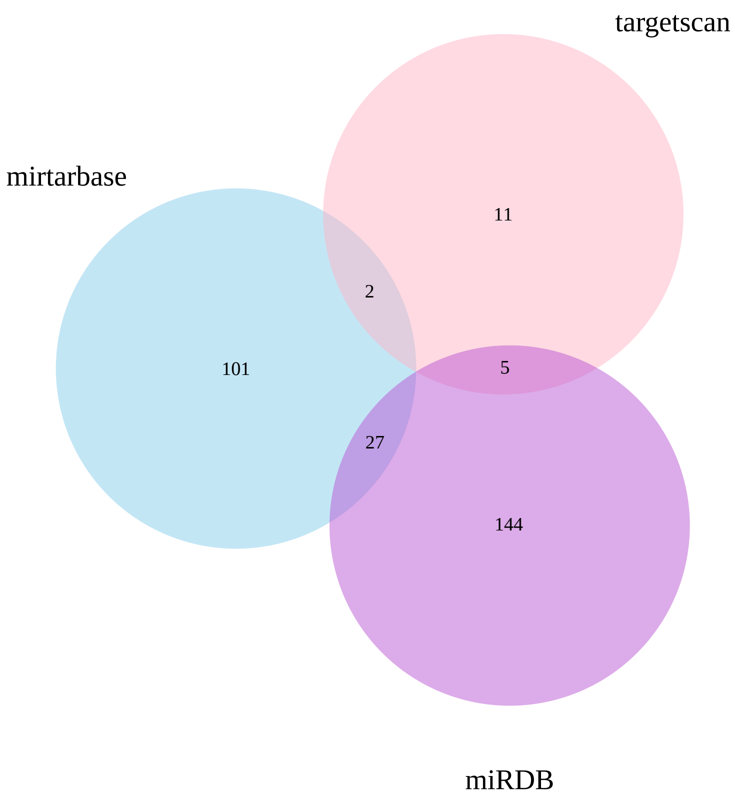


```
## (polygon[GRID.polygon.1365], polygon[GRID.polygon.1366], polygon[GRID.polygon.1367],
```
